# Astrocytic PYGM attenuates tau pathology by promoting lactate‐mediated neuroprotection

**DOI:** 10.1002/alz.71202

**Published:** 2026-02-17

**Authors:** Jing Cao, Jian Meng, Yiqing Chen, Ziqian Tang, Yong Wang, Kun Li, Xian Zhang, Hong Luo, Huihui Li, Zhanxiang Wang, Yun‐wu Zhang

**Affiliations:** ^1^ Xiamen Key Laboratory of Brain Center The First Affiliated Hospital of Xiamen University, Fujian Provincial Key Laboratory of Neurodegenerative Disease and Aging Research, Institute of Neuroscience, School of Medicine, Xiamen University Xiamen Fujian China; ^2^ Department of Neurosurgery and Department of Neuroscience The First Affiliated Hospital of Xiamen University, School of Medicine, Xiamen University Xiamen Fujian China; ^3^ Xiamen Neurosurgical Quality Control Center Xiamen Fujian China; ^4^ State Key Laboratory of Vaccines for Infectious Diseases Xiang An Biomedicine Laboratory Xiamen Fujian China

**Keywords:** astrocyte, glycogen metabolism, lactate, PYGM, tau, tauopathy

## Abstract

**INTRODUCTION:**

Tauopathies are characterized by hyperphosphorylated tau accumulation and neurodegeneration. Although astrocytic metabolism is known to support neuronal health, the role of astrocytic glycogen metabolism, particularly the glycogenolytic enzyme PYGM (glycogen phosphorylase, muscle associated), in tauopathies remains unclear.

**METHODS:**

We analyzed PYGM expression in the brains of frontotemporal lobar degeneration with ubiquitin‐positive inclusions (FTLD‐U) patients and tau^P301S^ transgenic (PS19) mice, assessed tauopathy‐related phenotypes in male PS19 mice with astrocyte‐specific PYGM knockout or overexpression, studied the effects of PYGM knockdown on astrocytes and neurons, and evaluated the effects of lactate supplementation on male PS19 mice.

**RESULTS:**

PYGM expression increased in the brains of FTLD‐U patients and the astrocytes of PS19 mice. Astrocytic PYGM deficiency impaired mouse cognition, exacerbated tauopathy‐related phenotypes in male PS19 mice, and disrupted astrocyte‐neuron metabolic coupling. PYGM overexpression and lactate supplementation attenuated tauopathy‐related phenotypes in male PS19 mice.

**DISCUSSION:**

Astrocytic PYGM supports neuronal health by sustaining lactate‐mediated astrocyte‐neuron metabolic coupling. Enhancing astrocytic glycogenolysis may be beneficial in tauopathies.

## Highlights

- PYGM (glycogen phosphorylase, muscle associated) expression increases in the brains of frontotemporal lobar degeneration with ubiquitin‐positive inclusions (FTLD‐U) patients and the tau^P301S^ transgenic (PS19) tauopathy model mice at pathological stages.
- Astrocytic PYGM deficiency impairs cognitive function in mice.
- Astrocytic PYGM deficiency exacerbates tauopathy‐related phenotypes and reduces lactate production in PS19 mice.
- Overexpression of PYGM in astrocytes and lactate supplementation confer protection in PS19 mice.

## 1 BACKGROUND

Tauopathies comprise a group of neurodegenerative disorders characterized by abnormal tau accumulation, including frontotemporal dementia (FTD), progressive supranuclear palsy (PSP), corticobasal degeneration (CBD), Alzheimer's disease (AD), etc.^1^, ^2^ Tau pathology disrupts neuronal microtubule stability, impairs synaptic communication, and triggers neuroinflammation, thereby culminating in neuronal death and brain atrophy, and finally leading to cognitive and/or motor deficits.^3^, ^4^ Although the majority of tauopathy research has focused on neuronal dysfunction, increasing evidence suggests that non‐neuronal cells also contribute significantly to disease progression.^5^, ^6^

Astrocytes are essential for maintaining central nervous system (CNS) homeostasis by supporting neuronal metabolism, modulating synaptic activity, maintaining the blood‐brain barrier, and regulating immune responses.^7^, ^8^, ^9^ In tauopathies, reactive astrogliosis is a common pathological hallmark, often associated with altered metabolic states and impaired neuroprotective functions.^10^, ^11^ Glycogen is a major energy reserve of the brain and predominantly stored in astrocytes. Astrocytes rapidly degrade glycogen into lactate and release it as an energy support in response to the request from neurons.^12^, ^13^, ^14^, ^15^ Glycogen metabolism also occurs in neurons and maintains neuronal health.^16^ Disturbance of glycogen metabolism can affect synaptic functions and impair cognition in animals,^17^, ^18^, ^19^ and also contribute to tau pathology. For example, glycogen synthase kinase‐3β (GSK3β) is a kinase participating in glycogen metabolism. GSK3β activity has been found to be dysregulated and can hyperphosphorylate tau under AD and other tauopathy background.^20^, ^21^ One recent study finds that tau can bind glycogen to promote glycogen accumulation in neurons, exacerbating tau accumulation and further disrupting cellular homeostasis, whereas breaking down neuronal glycogen ameliorates tauopathy‐related phenotypes in flies and iPSC‐derived neurons from people with frontotemporal lobar dementia (FTLD) ‐tau.^22^ Thus, glycogen dysregulation may drive the progression of tau‐mediated neurodegeneration. Despite these insights, the molecular mechanisms by which astrocytic glycogen influences tau pathology remain poorly understood. One emerging factor is PYGM (glycogen phosphorylase, muscle associated), a rate‐limiting enzyme in glycogen breakdown.^23^ Our recent study has linked neuronal PYGM to synaptic energy deficits and cognitive impairment in AD models.^19^ However, PYGM is enriched in astrocytes in the CNS.^24^, ^25^ Whether and how astrocytic PYGM participates in tauopathies has yet to be elucidated.

In the present study, we find that PYGM expression increases in the brains of patients with one tauopathic disorder, the frontotemporal lobar degeneration with ubiquitin‐positive inclusions (FTLD‐U), and in the astrocytes of the PS19 tauopathy mouse model at pathological stages. Astrocytic PYGM‐specific deletion impairs cognition in mice and exacerbates cognitive and motor deficits and tauopathy‐related pathologies in PS19 mice. In contrast, PYGM overexpression in astrocytes alleviates disease‐related phenotypes in PS19 mice. Mechanistic investigations reveal that astrocytic PYGM deficiency triggers intrinsic metabolic disturbances, characterized by glycogen accumulation, impaired lactate production and release, elevated reactive oxygen species (ROS), and increased pro‐inflammatory cytokine expression. These astrocytic alterations may subsequently impair energy metabolism and enhance tau phosphorylation in neurons. Finally, we show that lactate supplementation attenuates multiple tauopathy‐related phenotypes in PS19 mice. These findings highlight that astrocytic PYGM plays a critical neuroprotective role against tau pathology through mechanisms that include regulating lactate production to maintain energy homeostasis.

## 2 METHODS

### 2.1 Animals

*Pygm^flox/flox^* mice (C57BL/6J background, company number: CKOCMP‐19309‐Pygm‐B6J‐VA) were purchased from Cyagen. To achieve astrocyte‐specific *Pygm* deletion, *Pygm^flox/flox^* mice were crossed with *Aldh1l1*‐cre^ER^ mice (Jackson Laboratory, Stock No: 031008). The resultant *Pygm^flox/flox^*;*Aldh1l1*‐cre^ER^ mice were further crossed with PS19 mice (Jackson Laboratory, Stock No: 008169; expressing human mutant tau^P301S^) to generate four experimental groups: *Pygm^flox/flox^* (Ctrl), *Pygm^flox/flox^*;*Aldh1l1*‐cre^ER^ (AcKO), *Pygm^flox/flox^*;PS19 (PS19), and *Pygm^flox/flox^*;*Aldh1l1*‐cre^ER^;PS19 (AcKO;PS19). Cre‐mediated recombination was induced in 1‐month‐old mice via intraperitoneal tamoxifen (Sigma‐Aldrich, T5648) injection (75 mg/kg daily for 7 days). All mice were maintained under specific pathogen‐free conditions (22 ± 1°C, 12‐hour light/dark cycle) with free access to food and water. Animal experiments were approved by the Animal Ethics Committee of Xiamen University and conducted following the Committee's guidelines.

### 2.2 Cell cultures and treatments

Human embryonic kidney 293 cells (HEK293) were cultured in Dulbecco's modified Eagle's medium containing 10% fetal bovine serum and 1% penicillin/streptomycin (P/S). Mouse primary neurons and astrocytes were prepared and cultured as previously described.^26^ Astrocytes were maintained in culture medium for 3 days. Astrocyte‐conditioned medium (ACM) was then collected, and cellular debris was removed by centrifugation (1000 g, 10 minutes). In some experimental paradigms, primary neurons from wild‐type (WT) and PS19 mice were exposed to collected ACM for 3 days. In some other experiments, primary neurons from PS19 mice were cultured with the addition of 2 mM L‐lactate (Sigma‐Aldrich, 71718), 2 mM D‐glucose (TargetMol, T0887), or 2 mM oleic acid (TargetMol, T2O2668) for 3 days before the experiment.

### 2.3 Immunoblotting and antibodies

Proteins of brain tissues or cells were extracted into RIPA buffer, separated by sodium dodecyl sulfate—polyacrylamide gel electrophoresis (SDS‐PAGE), and transferred to polyvinylidene fluoride (PVDF) membranes. After blocking with 5% non‐fat milk, membranes were incubated overnight at 4°C with primary antibodies, followed by horseradish peroxidase (HRP) ‐conjugated secondary antibodies (LABLEAD, S0100 and S0101, 1:5000). Signals were detected using enhanced chemiluminescence (ECL) and quantified using ImageJ (NIH). Primary antibodies used were: anti‐α‐tubulin (PTM, 5442, 1:10000), anti‐β‐actin (Abclonal, AC026, 1:10000), anti‐Caspase‐3 (Cell Signaling Technology, 9662S, 1:1000), anti‐Cleaved‐Caspase‐3 (Cell Signaling Technology, 9664S, 1:1000), anti‐Cleaved‐PARP (Cell Signaling Technology, 9544S, 1:1000), anti‐GLAST (Proteintech, 20785‐1‐AP, 1:1000), anti‐GluA1 (Cell Signaling Technology, 13185S, 1:1000), anti‐GluA2 (Cell Signaling Technology, 13607S, 1:1000), anti‐GluN1 (Cell Signaling Technology, 5704S, 1:1000), anti‐GluN2A (Cell Signaling Technology, 4205S, 1:1000), anti‐GluN2B (Cell Signaling Technology, 14544S, 1:1000), anti‐GSK3β (Proteintech, 51065‐1‐AP, 1:1000), anti‐p‐GSK3β (Proteintech, 17517‐1‐AP, 1:1000), anti‐GYS1 (4A biotech, 4ab086436, 1:1000), anti‐PARP (Cell Signaling Technology, 9532S, 1:1000), anti‐PSD93 (Abcam, ab94588, 1:1000), anti‐PSD95 (Cell Signaling Technology, 3450S, 1:1000), anti‐PYGB (4A biotech, 4ab093844, 1:1000), anti‐PYGL (4A biotech, 4ab094049, 1:1000), anti‐PYGM (Abcam, ab231963, 1:1000), anti‐p‐Tau T181 (Cell Signaling Technology, 12885S, 1:1000), anti‐p‐Tau S199 (Invitrogen, 44734G, 1:1000), anti‐p‐Tau T205 (Biosource, 44‐738G, 1:1000), anti‐p‐Tau T212 (Biosource, 44‐740G, 1:1000), anti‐p‐Tau T231/S235 (Cell Signaling Technology, 20473S, 1:1000), anti‐p‐Tau S396 (Invitrogen, 44752G, 1:1000), anti‐p‐Tau S404 (Cell Signaling Technology, 20194S, 1:1000), and anti‐total tau (Invitrogen, AHB0042, 1:1000). Original immunoblot data are provided in Figure S1.

**RESEARCH IN CONTEXT**

1. **Systematic review**: We reviewed prior studies examining astrocytic metabolism in neurodegeneration. Evidence shows that enhanced glycolysis and lactate production in astrocytes support cognition and neuronal survival in Alzheimer's models. However, the role of glycogen breakdown, particularly via PYGM (glycogen phosphorylase, muscle associated)‐mediated glycogen degradation in tauopathy, remains unexplored.
2. **Interpretation**: Our findings demonstrate that astrocytic PYGM is upregulated in tauopathy and is essential for cognitive function, synaptic integrity, and neuronal survival. Conditional deletion of PYGM in astrocytes leads to energy deficits, gliosis, and exacerbated tau pathology, whereas its overexpression is protective. Lactate emerges as an important mediator of astrocytic PYGM‐exerted neuroprotection. This work identifies an essential role of astrocytic PYGM in sustaining energy homeostasis during tauopathy.
3. **Future directions**: PYGM‐regulated astrocytic glycogen metabolism may represent a novel therapeutic target in tauopathy. To elucidate the underlying mechanisms and determine the feasibility of such a therapeutic strategy, additional studies are required and examples include: (1) revealing the regulatory mechanisms controlling astrocytic PYGM expression during tauopathy; (2) identifying the signaling pathways by which astrocytic PYGM activity influences glial inflammatory responses, neuronal survival, and tau phosphorylation; (3) determining whether systemic metabolic modulators (e.g. PYGM activators) can safely augment astrocytic glycogen metabolism in vivo; (4) exploring whether similar mechanisms are operative in diverse tauopathies, such as progressive supranuclear palsy (PSP) and corticobasal degeneration (CBD).

### 2.4 Immunofluorescence staining

Brain sections were washed in 1× phosphate‐buffered saline (PBS) for 15 minutes, blocked with 5% bovine serum albumin (BSA) and 0.2% Triton X‐100 in PBS for 1 hour at room temperature, and then incubated with primary antibodies overnight at 4°C. After PBS washing, sections were incubated with species‐matched Alexa Fluor‐conjugated secondary antibodies for 1 hour at room temperature. The nuclei were counterstained with 4′,6‐diamidino‐2‐phenylindole (DAPI) for 10 minutes, and the slides were mounted with anti‐fade reagent (Boster Biological Technology, AP1109). Images were acquired using a confocal microscope and analyzed with ImageJ (NIH). Antibodies used were: anti‐glial fibrillary acidic protein (GFAP; Proteintech, 16825‐1‐AP, 1:300), anti‐Iba1 (Wako, 019‐19741, 1:300), anti‐NeuN (Cell Signaling Technology, 24307S, 1:300), anti‐PYGM (Invitrogen, MA527442, 1:200), anti‐p‐Tau T231/S235 (Cell Signaling Technology, 20473S, 1:200), and secondary antibodies (Thermo Fisher Scientific, A‐11008; A‐11001; A‐11012; A‐11005, all 1:400).

### 2.5 TUNEL staining

Terminal deoxynucleotidyl transferase dUTP Nick‐End Labeling (TUNEL) staining was performed using a fluorescein isothiocyanate (FITC) Apoptosis Detection Kit (Vazyme, Cat# A111‐01) following the manufacturer's protocol. Briefly, brain sections were blocked with 5% BSA and 0.2% Triton X‐100 in PBS for 1 hour at room temperature. Sections were equilibrated with the equilibration buffer (provided in the kit) for 20 minutes at room temperature, followed by incubation with terminal deoxynucleotidyl transferase (TdT) reaction mixture, which covered the tissue area in a wet box, at 37°C for 1 hour. After three 5‐minute washes with PBS, the sections were subjected to fluorescence staining.

### 2.6 AAV and lentiviral production

AAV2/9 serotype viruses were generated via triple plasmid co‐transfection in HEK293T cells using AAV transfer plasmids expressing *GfaABC1D‐P2A‐mCherry* or *GfaABC1D‐PYGM‐HA‐P2A‐mCherry*, the pAd‐deltaF6 helper plasmid (Addgene, #112867), and the pAAV2/9 packaging plasmid (Addgene, #112865). Viral particles were harvested 72 hours post‐transfection and purified by iodixanol gradient ultracentrifugation. The AAV plasmids were kindly provided by Dr. Yingjun Zhao at Xiamen University.

*Pygm*‐targeting shRNAs (designed via the Thermo Scientific™ TRC Lentiviral™ shRNA Library) were cloned into the pLKO.1 plasmid (provided by Dr. Qinxi Li at Xiamen University). HEK293T cells were co‐transfected with shRNA plasmids, the psPAX2 packaging plasmid, and the pMD2.G envelope plasmid. Lentiviral supernatants were collected 48 hours post‐transfection. The sequences for negative control (shNC) are: Sense: 5′‐CCTAAGGTTAAGTCGCCCTCG‐3′, and Antisense: 5′‐ CGAGGGCGACTTAACCTTAGG‐3′. The sequences for shPygm are: Sense: 5′‐CTATCGGAAACAACGTCGTCAA‐3′, and Antisense: 5′‐TTGACGACGTTGTTCCGATAG‐3′.

### 2.7 Stereotaxic injections

For astrocytic PYGM overexpression in the mouse hippocampus, 6‐month‐old mice were anesthetized and immobilized in a stereotaxic frame. AAV2/9 viruses (titer: 2.00×10^1^ ^2^ vg/mL) were bilaterally injected (1 µL per hemisphere) into hippocampal coordinates (anteroposterior [AP]: −2.0 mm; mediolateral [ML]: ± 1.5 mm; dorsoventral [DV]: 1.8 mm).

For sparse labeling of hippocampal pyramidal neurons, 9‐month‐old mice were anesthetized and immobilized in a stereotaxic frame. The rAAV‐hSyn‐FLP‐WPRE‐hGH pA viruses (titer: 5.60×10^1^ ^2^ vg/mL, purchased from BrainVTA) were first diluted at 1:20,000 in sterile PBS, and then mixed at a 1:1 volume ratio with rAAV‐nEF1α‐FDIO‐EYFP‐EYFP‐WPRE‐hGH pA (titer: 5.35×10^1^ ^2^ vg/mL, purchased from BrainVTA). The viral cocktail was bilaterally injected (200 nL per hemisphere) into hippocampal coordinates (AP: −2.0 mm; ML: ± 1.0 mm; DV: 1.8 mm).

For L‐lactate infusion via intracranial cannulation, 9‐month‐old WT or PS19 male mice were implanted with stereotaxic cannulas targeting the lateral ventricle (coordinates relative to bregma: AP: −0.3 mm; ML: −1.0 mm; DV: −2.1 mm). After a 1‐week recovery period, 1 µL of L‐lactate (200 nmol) was infused into the lateral ventricle at a rate of 0.2 µL/min. Behavioral tests were conducted 24 hours after the infusion.

### 2.8 Cerebrospinal fluid collection

Mice were anesthetized and immobilized in a stereotaxic adapter with the dorsal skull angled at ∼45°. The skin over the occipital region was incised midline, and the subcutaneous tissues were pulled apart to expose the cisterna magna. Under a dissecting microscope, residual musculature and connective tissues were carefully dissected using fine forceps. Hemostasis was achieved with sterile cotton swabs. The dura was cleansed with saline‐moistened swabs and dried. A glass capillary with a sharpened tip was advanced through the dura at a 30–45° angle relative to the skull base under microscopic guidance. Cerebrospinal fluid (CSF) was collected via capillary action into the tube and stored at −80°C.

### 2.9 Behavioral analysis

Only male mice were subjected to behavioral analysis. Prior to tests, mice were acclimated to the testing environment for 30 minutes. All experiments were conducted by investigators blinded to the experimental groups. Behavioral data were acquired and analyzed using TopScan Lite software (Clever Systems Inc.) following established protocols.^26^

#### 2.9.1 Y‐maze test

Mice were allowed to freely explore a Y‐maze (three identical arms at 120° angles) for 5 minutes. Spontaneous alternation percentage was calculated as (number of consecutive entries into three different arms / [total arm entries − 2]) × 100.

#### 2.9.2 Morris Water Maze test

The Morris Water Maze test was conducted in a circular pool (120 cm diameter) containing opaque water (temperature: 20°C –22°C), surrounded by four distinct visual cues. During a 5‐day training phase, mice underwent two daily trials (each lasting 60 seconds) to locate a submerged platform positioned in a fixed target quadrant. Unsuccessful mice were guided to the platform and allowed to stay there for 10 seconds. A probe testing trial was subsequently performed by removing the platform and allowing 60 seconds of free exploration. Analyzed parameters included: escape latency during the training phase, and average swim speed and number of platform area crossings during the probe testing phase.

#### 2.9.3 Fear conditioning test

Mice were first acclimated for 2 minutes in Chamber A (metal grid floor). Three conditioning trials were delivered at 1‐minute intervals, each consisting of a 30‐second conditioned stimulus (CS: 75‐dB white noise) that co‐terminated with a 2‐second 0.8‐mA scrambled foot shock (unconditioned stimulus, US). Contextual memory was assessed by a 5‐minute re‐exposure to Chamber A without stimuli. Cued memory was tested in Chamber B (cylindrical, 20×30 cm) with a 3‐minute baseline followed by a 3‐minute 75‐dB CS presentation. Mouse freezing was quantified in both phases.

#### 2.9.4 Limb clasping

Mice were gently suspended by the tail for 10 seconds to record their full‐body limb movements. The limb clasping was scored using standardized criteria.^27^

### 2.10 Quantitative real‐time polymerase chain reaction

RNA processing and analysis followed established protocols.^28^ Target‐specific primer sequences were as follows: *Pygm*, Forward‐5′‐AGCTGGAGCCTCACAAGTTC‐3′, and Reverse‐5′‐CTGGACGTCAAAGAGCGAGT‐3′; *Gys1*, Forward‐5′‐ATCTACACTGTGCTGCAGACG‐3′, and Reverse‐5′‐CCCTTGCTGTTCATGGAATCC‐3′; *Gys2*, Forward‐5′‐CCATCCTCAGCACCATTAGAC‐3′, and Reverse‐5′‐GTGACAACCTCGGACAAACTC‐3′; *Pygl*, Forward‐5′‐TGCTTTGGATAAGAAGGGGTATGAGGC‐3′, and Reverse‐5′‐TTGAAGAGGTCTGGCTGATTGGGAG‐3′; *Pygb*, Forward‐5′‐CAGCAGCATTACTATGAGCGG‐3′, and Reverse‐5′‐CAGCAGCATTACTATGAGCGG‐3′; *Ppp1R3C*, Forward‐5′‐TGATCCATGTGCTAGATCCACG‐3′, and Reverse‐5′‐ACTCTGCGATTTGGCTTCCTG‐3′; and *Gyg*, Forward‐5′‐ACACCTTCACCACCAACGTCTT‐3′, and Reverse‐5′‐GCTCCTGAGACATGTTCCATCAT‐3′.

### 2.11 Lactate quantification

Lactate concentrations were determined using a commercial enzymatic assay kit (Elabscience, Cat# E‐BC‐K044‐M), following the manufacturer's protocol. Briefly, cell lysate, CSF, or ACM samples were mixed with a reaction buffer containing lactate oxidase and peroxidase, and incubated at 37°C for 10 minutes. The absorbance was read at 530 nm using a microplate reader.

### 2.12 Glycogen measurement

Glycogen content was measured using a commercial enzymatic assay kit (Solarbio, Cat# BC0345), following the manufacturer's protocol. Briefly, cells were lysed in alkaline extraction buffer, incubated in a boiling water bath for 20 minutes to hydrolyze glycogen, and centrifuged at 8000 g for 10 minutes to collect supernatants. Supernatants were mixed with anthrone reagent (in H_2_SO_4_), incubated in a boiling water bath for 10 minutes, and cooled on ice. The absorbance was measured at 620 nm.

### 2.13 Periodic acid Schiff staining

Glycogen was analyzed using periodic acid Schiff (PAS) histochemical staining with a commercial kit (Solarbio, Cat# G1360), following the manufacturer's protocol. Briefly, cells were oxidized with 0.5% periodic acid to expose glycogen aldehyde groups, incubated with Schiff reagent for 15 minutes to generate magenta‐colored complexes, and counterstained with hematoxylin to visualize the nuclei. The staining intensity from microscopic images was subjected to digital quantification using ImageJ (NIH).

### 2.14 Electrophysiological recording

Electrophysiology was performed as previously described.^26^ Briefly, mice were anesthetized with isoflurane and decapitated. Mouse brains were rapidly removed and placed into ice‐cold oxygenated cutting solution. Hippocampal slices (400 µm) were prepared using a vibratome, incubated in oxygenated ACSF at 32°C for 1 hour, and equilibrated at room temperature. During recording, slices were perfused with artificial CSF (ACSF; 2 mL/min). A stimulating electrode was placed in the CA3, and a recording electrode (0.5–2 MΩ) was placed in the CA1 to monitor field excitatory postsynaptic potentials (fEPSPs). Baseline stimulation intensity was determined by generating stimulus‐response curves (10–700 mA range) and selecting the current that elicited 30% of the maximal fEPSP amplitude. Baseline responses were recorded for 20 minutes. Long‐term potentiation (LTP) was induced by high‐frequency stimulation (2 trains, 100 Hz, 30‐second interval) and monitored for 60 minutes. Data were sampled at a rate of 10 kHz and filtered at 2 kHz using Multiclamp 700B amplifier and analyzed with pClamp 10.6 software (Molecular Devices).

### 2.15 Extracellular acidification rate and oxygen consumption rate measurements

Cellular glycolytic (extracellular acidification rate [ECAR]) and mitochondrial (oxygen consumption rate [OCR]) activities were quantified using a Seahorse XFe96 Analyzer (Agilent Technologies). Astrocytes or neurons (5 × 10^4^ cells/well) were seeded in XF96 cell culture microplates and preconditioned in ECAR medium (Agilent Technologies, Cat# 103577‐100) or OCR medium (Agilent Technologies, Cat# 103575‐100), supplemented with 1 mM sodium pyruvate (Agilent Technologies, Cat# 103578‐100), 2 mM glutamine (Agilent Technologies, Cat# 103579‐100), and 10 mM D‐glucose (Agilent Technologies, Cat# 103577‐100). Following 1 hour equilibration in a non‐CO_2_ environment, baseline measurements were acquired prior to sequential injection of metabolic modulators: for ECAR assays, 500 mM 2‐DG and 1 µM rotenone/antimycin A were used; and for OCR assays, 1 µM oligomycin, 0.5 µM FCCP, and 1 µM rotenone/antimycin A were used.

### 2.16 Flow cytometric detection of ROS

To measure ROS levels, primary astrocytes subjected to PYGM knockdown (shPygm) and controls (shNC) were trypsinized, centrifuged (1200 g, 5 minutes), and incubated with 10 µM 2′‐7′‐dichlorodihydrofluorescein diacetate (DCFH‐DA, Beyotime, S1105S) in serum‐free medium at 37°C for 30 minutes in darkness. After two washes with ice‐cold PBS, cells were resuspended in 500 µL of ice‐cold PBS and analyzed immediately by flow cytometry.

### 2.17 Statistics

Data were analyzed using GraphPad Prism 9 software and presented as mean ± SEM. For comparisons between two groups, a two‐tailed unpaired Student's *t*‐test was applied. For multiple comparisons, one‐way analysis of variance (ANOVA) with Tukey's post hoc test and two‐way ANOVA with Tukey's post hoc test or Dunn‐Šídák post hoc test were applied. Statistical significance was defined as follows: *p < 0.05, **p < 0.01, ***p < 0.001, and ****p < 0.0001. Specific method details are provided in the corresponding figure legends. A post‐hoc power analysis was carried out using G*Power to evaluate the achieved statistical power for the primary endpoints based on animal numbers used for behavioral analyses, and the results are presented in Table S1.

## 3 RESULTS

### 3.1 PYGM expression increases in the brains of tauopathy patients and the astrocytes of PS19 mice at pathological stages

**FIGURE 1:**
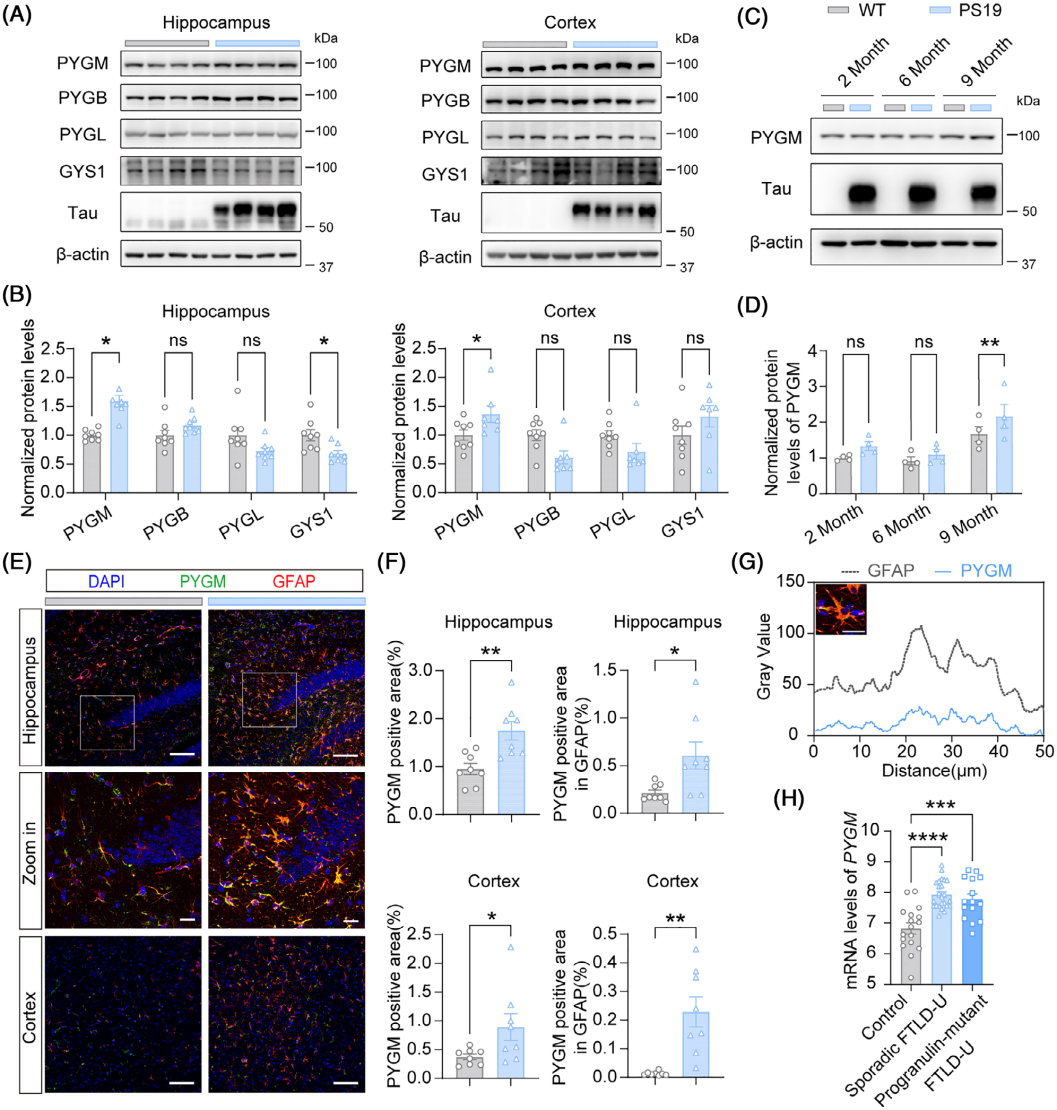
Elevated PYGM (glycogen phosphorylase, muscle associated) expression in the astrocytes of tau^P301S^ transgenic (PS19) mice at pathological stages and the brains of frontotemporal lobar degeneration with ubiquitin‐positive inclusions (FTLD‐U) patients. (A, B) Immunoblot (A) and quantitative analysis (B) of PYGM, PYGB, PYGL, and GYS1 protein levels in the hippocampus and cortex of 9‐month‐old male PS19 mice and wild‐type (WT) littermates. *n* = 8 per group. (C, D) Immunoblot (C) and quantitative analysis (D) of hippocampal PYGM protein levels in 2‐, 6‐, and 9‐month‐old male PS19 and WT littermates. *n* = 4 per group. (E, F) Immunofluorescence staining of PYGM (green) and the astrocytic marker glial fibrillary acidic protein (GFAP) (red) in the hippocampus and cortex of 9‐month‐old PS19 mice and WT littermates (E). PYGM‐positive area and PYGM colocalization with GFAP were quantified for comparison (F). The nuclei were counterstained with 4′,6‐diamidino‐2‐phenylindole (DAPI; blue). Scale bars: 100 µm for regular and 20 µm for zoom‐in images. *n* = 8 per group. (G) Line scan analysis of PYGM and GFAP colocalization. The graph shows fluorescence intensity profiles of PYGM (blue) and GFAP (gray) along the distance of the rectangular region in the inset, indicating their spatial relationship. Scale bar: 20 µm. (H) Comparison of *PYGM* mRNA levels in post‐mortem brain tissues of FTLD‐U patients with progranulin gene mutations (progranulin‐mutant FTLD‐U, *n* = 15), FTLD‐U patients without progranulin gene mutations (sporadic FTLD‐U, *n* = 24), and healthy controls (control, *n* = 17) from the GSE13162 dataset. Data are presented as mean ± SEM. *p* values were determined by two‐tailed unpaired Student's *t* test in (B and F), two‐way analysis of variance (ANOVA) followed by Dunn‐Šídák post hoc analysis in (D), and one‐way ANOVA followed by Tukey's post hoc analysis in (H). ns, not significant; *p < 0.05; **p < 0.01; ***p < 0.001; ****p < 0.0001.

To investigate the potential role of glycogen metabolism in tauopathies, we first analyzed the protein levels of PYGM, PYGB, and PYGL, which degrade glycogen, and GYS1, which synthesizes glycogen, in the hippocampus and cortex of 9‐month‐old male and female PS19 mice. Compared to WT littermates, male PS19 mice had significantly elevated PYGM levels in both the hippocampus and cortex, and decreased GYS1 levels only in the hippocampus. No significant changes were observed for other enzymes in the hippocampus or cortex (Figure 1A, B). In contrast, female PS19 mice had significantly elevated levels of PYGM and PYGB and decreased levels of GYS1 only in the hippocampus, and no significant changes in the cortex (Figure S2A, B). Since PYGM is the only enzyme consistently increased in both the hippocampus and cortex of male PS19 mice, we focused on studying PYGM in tauopathy using male mice in the present study. Notably, hippocampal PYGM levels were unaltered in PS19 mice at 2 and 6 months of age (Figure 1C, D), suggesting that its upregulation is specific to the later disease stage when tau pathologies and behavioral deficits manifest.

Immunofluorescence analysis revealed predominant colocalization of PYGM with the astrocytic marker GFAP but not the neuronal marker NeuN or the microglial marker Iba1 (Figure 1E, 1G, and S2C‐F), suggesting that PYGM is mainly expressed in astrocytes. Both the fluorescence intensity of PYGM and its colocalization with GFAP significantly increased in the hippocampus and cortex of 9‐month‐old PS19 mice (Figure 1E, F). Moreover, we noticed that in one study comparing gene expression of brain tissues from FTLD‐U patients with or without progranulin mutations and control cases (Accession number GEO: GSE13162),^29^ PYGM expression was significantly elevated in both FTLD‐U patients with and without progranulin mutations compared to controls (Figure 1H). In contrast, PYGB and PYGL levels remained unchanged, and GYS1 levels decreased in FTLD‐U patients (Figure S2G). Collectively, these results from both mouse models and human data suggest that astrocytic PYGM may play a crucial role in tauopathy at pathological stages.

### 3.2 Deficiency of astrocytic PYGM impairs cognitive function in mice and exacerbates cognitive and motor deficits associated with tauopathy

To investigate the role of astrocytic PYGM in cognitive function, we crossed *Pygm^flox/flox^* mice with *Aldh1l1*‐cre^ER^ mice. The offspring, including *Pygm^flox/flox^* (Ctrl) mice and *Pygm^flox/flox^*;*Aldh1l1*‐cre^ER^ (AcKO) mice, were administered with tamoxifen at 1 month of age to induce astrocyte‐specific *Pygm* ablation in astrocyte‐specific conditional knockout (AcKO) mice. Mice were then assessed for their behaviors at 3 months of age (Figure S3A). About 50% of PYGM reduction in the hippocampus and in astrocytes of AcKO mice was found by immunoblotting and immunofluorescence analyses, respectively (Figure S3B‐E). Such a partial reduction likely reflects an incomplete recombination mediated by the *Aldh1l1*‐Cre^ER^ system upon tamoxifen induction in vivo, and this phenomenon has also been observed in other studies.^30^, ^31^, ^32^ Nevertheless, we found that compared to Ctrl mice, AcKO mice exhibited impairments in spatial learning and memory, as evidenced by prolonged escape latencies during the training phase and fewer platform crossings during the probe testing phase in the Morris Water Maze test (Figure S3F, G). There was no swimming speed difference between AcKO and Ctrl mice (Figure S3H). Furthermore, AcKO mice showed significantly reduced spontaneous alternation in the Y‐maze test (Figure S3I), suggesting impaired short‐term working memory. However, there were no differences between AcKO and Ctrl mice in their freezing percentage in the cued and contextual fear conditioning tests (Figure S3J‐K). Together, these results suggest that astrocytic PYGM deletion impairs certain cognitive functions in mice.

**FIGURE 2:**
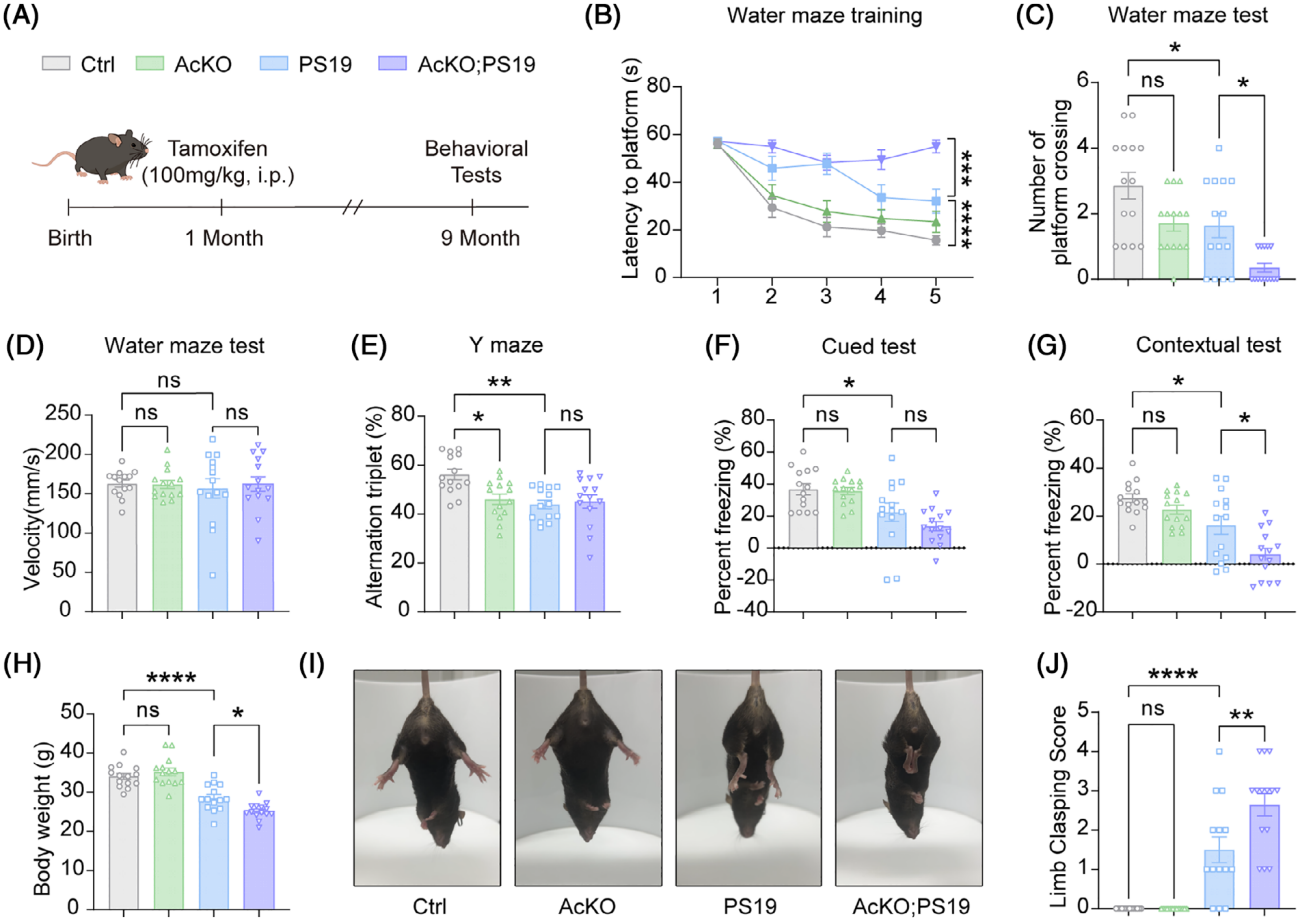
Astrocytic PYGM (glycogen phosphorylase, muscle associated) ablation exacerbates behavioral deficits in tau^P301S^ transgenic (PS19) mice. **(A)** Experimental timeline for tamoxifen administration and behavioral tests. Four genotypes of mice were studied: *Pygm^flox/flox^* (Ctrl), *Pygm^flox/flox^*;*Aldh1L1*‐cre^ER^ (AcKO), *Pygm^flox/flox^*;PS19 (PS19), and *Pygm^flox/flox^*;*Aldh1L1*‐cre^ER^;PS19 (AcKO; PS19). (B‐D) Mice were subjected to the Morris Water Maze test to compare the escape latency to reach the platform during a 5‐day training phase (B), and the number of platform region crossings (C) and the average swimming speed (D) during the probe test. (E) Mice were studied for their spontaneous alternations in the Y‐maze test. (F, G) Mice were subjected to the fear conditioning test to compare the percentage of freezing time in the cued (F) and contextual (G) phases. (H) The body weights of mice were compared. (I, J) Representative images of the hindlimb clasping of different mice (I) and the comparison of hindlimb clasping scores (J). Data are presented as mean ± SEM. *p* values were determined by two‐way analysis of variance (ANOVA) followed by Tukey's post hoc analysis in (B), and one‐way ANOVA followed by Tukey's post hoc analysis in (C‐H, and J). *n* = 14 mice per group for all these tests. ns, not significant; *p < 0.05; **p < 0.01; ***p < 0.001; ****p < 0.0001.

To further assess the role of astrocytic PYGM in tauopathies, we crossed *Pygm* conditional knockout mice with PS19 mice to generate four experimental groups: *Pygm^flox/flox^* (Ctrl), *Pygm^flox/flox^*;*Aldh1l1*‐cre^ER^ (AcKO), *Pygm^flox/flox^*;PS19 (PS19), and *Pygm^flox/flox^*;*Aldh1l1*‐cre^ER^;PS19 (AcKO;PS19). These mice were administered with tamoxifen at 1 month of age and studied for their behaviors at 9 months of age (Figure 2A). In the Morris Water Maze test, AcKO;PS19 mice exhibited significantly longer escape latencies during the training phase and fewer platform crossings during the probe testing phase than PS19 mice, though their swimming speeds were not altered (Figure 2B–D). In the Y‐maze test, PS19 mice displayed reduced spontaneous alternation compared to Ctrl mice, whereas no significant difference was observed between PS19 and AcKO;PS19 mice (Figure 2E). In the fear conditioning test, PS19 mice exhibited reduced freezing time in both the cued and contextual tests, and AcKO;PS19 mice had more contextual freezing than PS19 mice (Figure 2F, G). Moreover, we found that AcKO;PS19 mice exhibited more severe physical deterioration, including greater body weight loss and worse hindlimb clasping than PS19 mice (Figure 2H–J). Taken together, these findings demonstrate that the ablation of astrocytic PYGM exacerbates both cognitive decline and motor dysfunction in the context of tauopathy.

### 3.3 Deficiency of astrocytic PYGM aggravates tauopathy‐related pathologies in PS19 mice

**FIGURE 3:**
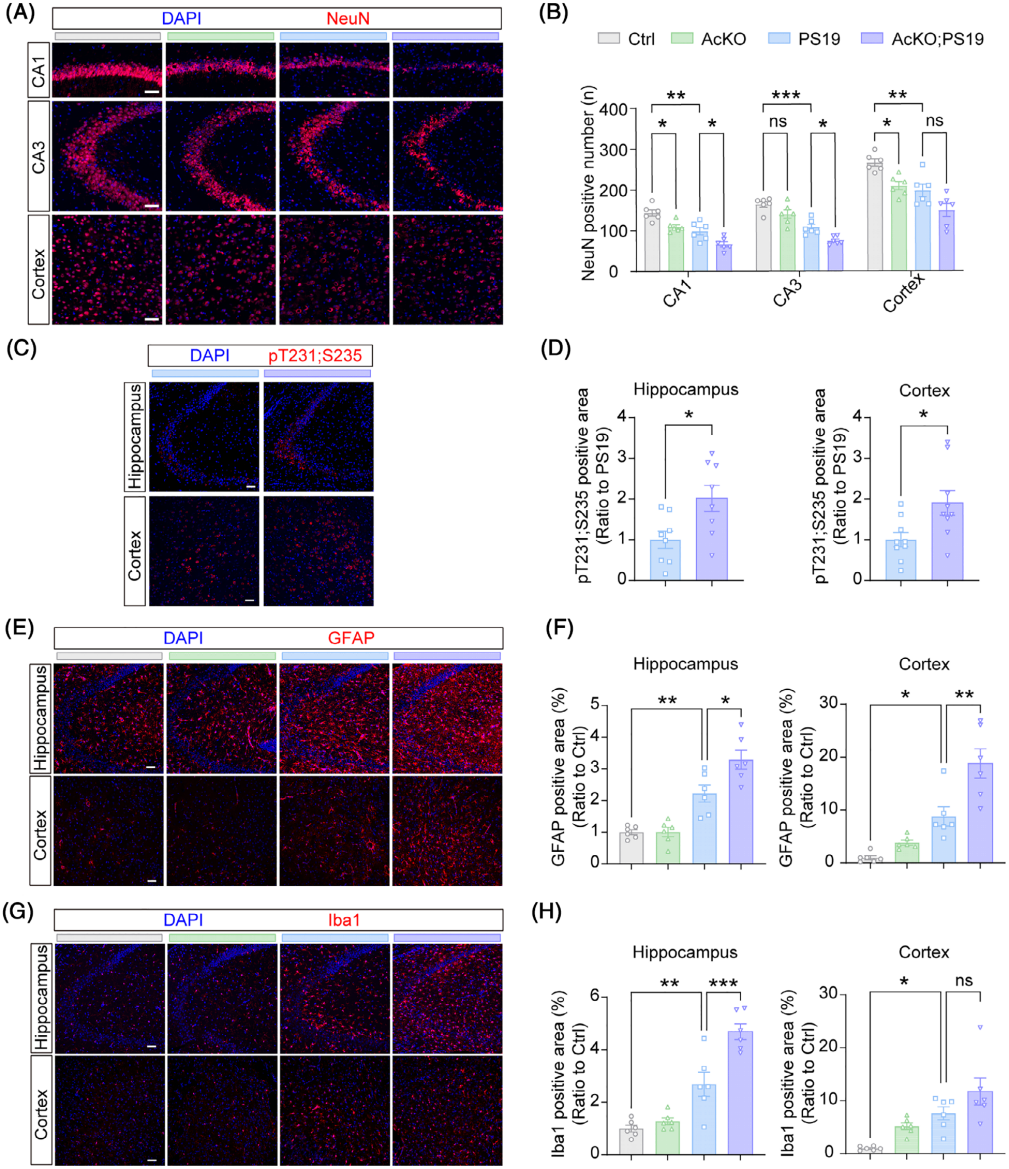
Astrocytic PYGM (glycogen phosphorylase, muscle associated) ablation exacerbates tauopathy‐related pathologies in tau^P301S^ transgenic (PS19) mice. (A, B) Immunofluorescence staining of NeuN (red) (A) and quantitative analysis (B) in brain sections from different mice. The nuclei were counterstained with 4′,6‐diamidino‐2‐phenylindole (DAPI; blue). Scale bars: 50 µm. *n* = 6 per group. (C, D) Immunofluorescence staining of phosphorylated tau at sites T231/S235 (red) (C) and quantitative analysis (D) in brain sections from different mice. The nuclei were counterstained with DAPI (blue). Scale bars: 100 µm. *n* = 8 per group. (E, F) Immunofluorescence staining of glial fibrillary acidic protein (GFAP; red) (E) and quantitative analysis (F) in brain sections from different mice. The nuclei were counterstained with DAPI (blue). Scale bars: 100 µm. *n* = 6 per group. (G, H) Immunofluorescence staining of Iba1 (red) (G) and quantitative analysis (H) in brain sections from different mice. The nuclei were counterstained with DAPI (blue). Scale bars: 100 µm. *n* = 6 per group. Data are presented as the mean ± SEM. *p* values were determined by one‐way analysis of variance (ANOVA) followed by Tukey's post hoc analysis in (B, F, and H), and two‐tailed unpaired Student's *t* test in (D). ns, not significant; *p < 0.05; **p < 0.01; ***p < 0.001.

PS19 mice at pathological stages exhibit impaired synaptic plasticity, synaptic integrity, and neuronal loss.^33^ To evaluate neuronal loss, we quantified NeuN‐positive cells in the mouse hippocampal CA1 and CA3 regions and cortex after behavioral tests. PS19 mice showed significant neuronal loss across all three regions compared to Ctrl mice; and AcKO;PS19 mice displayed aggravated neuronal loss in both CA1 and CA3 regions compared to PS19 mice (Figure 3A, B). TUNEL staining also revealed a significant increase in apoptotic cells in the hippocampus of AcKO;PS19 mice compared to PS19 mice (Figure S4A, B). Moreover, we found that AcKO mice exhibited reduced neuronal numbers in CA1 and cortical regions compared to Ctrl mice (Figure 3A, B), suggesting that astrocytic deletion of PYGM alone can cause neuronal loss.

We next examined the expression of excitatory synaptic proteins. Immunoblotting revealed significantly decreased levels of NMDA receptor subunits (GluN2B, GluN1), AMPA receptor subunits (GluA1, GluA2), and postsynaptic density proteins (PSD95, PSD93) in PS19 mice compared to Ctrl mice (Figure S4C, D). Astrocytic PYGM deletion further reduced the protein levels of GluN2B and GluA1 in the PS19 background (AcKO;PS19). Electrophysiological and structural analyses confirmed that 9‐month‐old PS19 mice exhibited impaired LTP and reduced dendritic spine density in the hippocampus compared to Ctrl mice (Figure S4E–H). However, AcKO;PS19 mice did not show further exacerbation of LTP impairment or spine loss compared to PS19 mice (Figure S4C–F). One possible reason is that the LTP impairment and spine loss driven by tauopathy in PS19 mice may already be saturated at this stage, limiting the detectability of further impairment caused by astrocytic PYGM loss. On the other hand, AcKO mice displayed significant deficits in both LTP and spine density compared to Ctrl mice (Figure S4E–H), suggesting that astrocytic PYGM deficiency alone can impair synaptic functions.

Tau hyperphosphorylation is a critical pathological hallmark of tauopathy. Immunofluorescence staining of phosphorylated tau at sites T231/S235 in the hippocampus and cortex showed that astrocytic PYGM deletion increased tau phosphorylation (Figure 3C, D). Consistently, immunoblot analysis revealed that astrocytic PYGM deletion significantly enhanced tau phosphorylation at multiple sites (S396, S404, S199, T231/S235, and T181) without altering total tau levels in the PS19 background (Figure S4I, J).

Moreover, immunofluorescence analysis revealed that both GFAP (Figure 3E, F) and Iba1 (Figure 3G, H) intensities significantly increased in the hippocampus and cortex of AcKO;PS19 mice compared to PS19 mice. These results indicate that astrocytic PYGM deletion exacerbates tauopathy‐associated astrogliosis and microgliosis.

Altered expression of excitatory amino acid transporters such as GLAST (also known as EAAT1) can cause glutamate excitotoxicity to drive neuronal death.^34^, ^35^ We found that GLAST expression was significantly increased in PS19 mice compared to Ctrl mice, but was not affected by PYGM deletion (Figure S4K, L). Additionally, we assessed the activity of GSK3β, a kinase participating in both glycogen metabolism and tau phosphorylation. Although the active form of GSK3β (pT216‐GSK3β) was found elevated in PS19 mice, such an elevation was not reversed by astrocytic PYGM deletion (Figure S4M, N). Taken together, these findings highlight a neuroprotective role of astrocytic PYGM in mitigating synaptic compromise, tau hyperphosphorylation, gliosis, and neuronal loss in tauopathy, likely through mechanisms independent of GLAST and GSK3β regulation.

### 3.4 Astrocytic PYGM overexpression alleviates cognitive and motor deficits in PS19 mice

**FIGURE 4:**
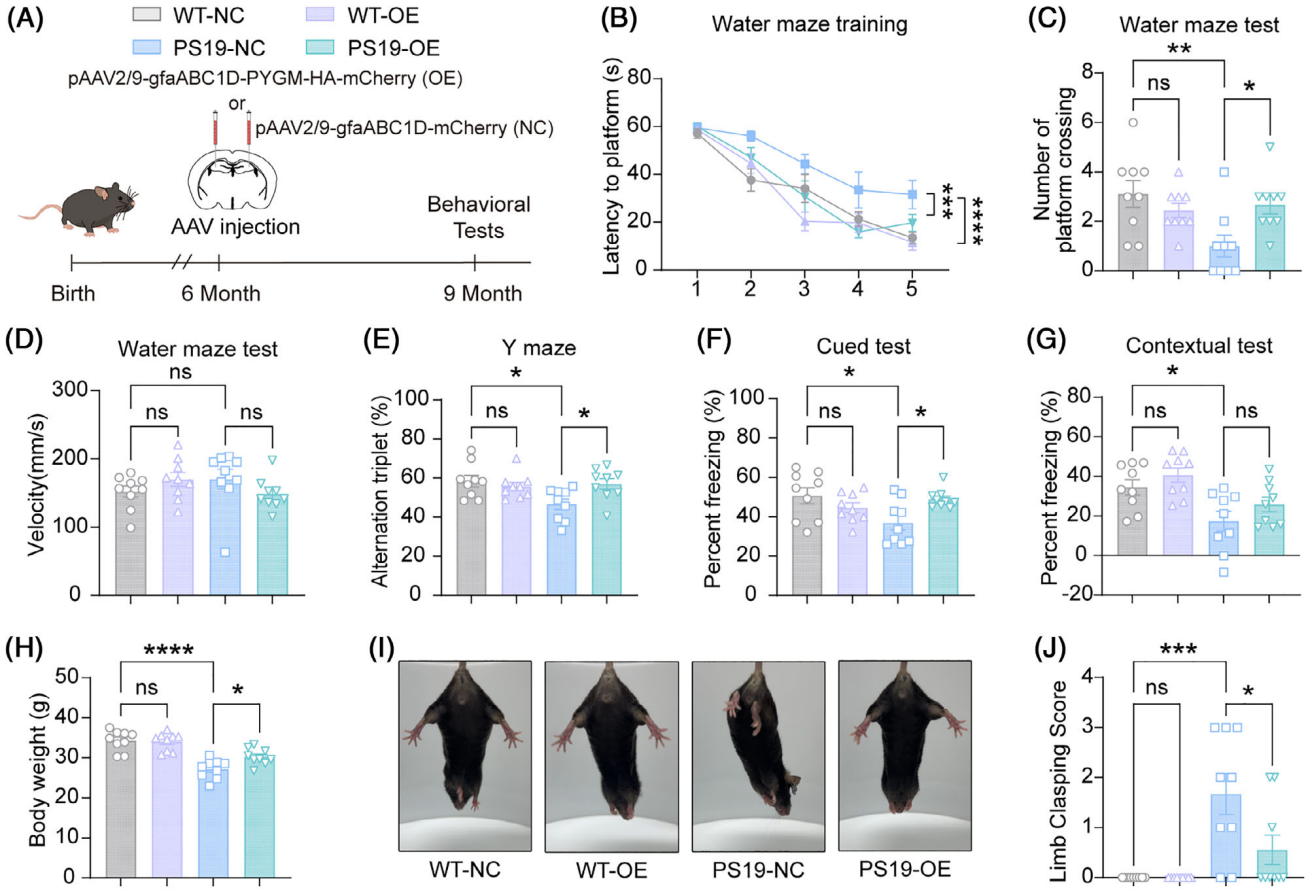
Astrocytic PYGM (glycogen phosphorylase, muscle associated) overexpression attenuates cognitive and motor deficits in tau^P301S^ transgenic (PS19) mice. (A) Experimental timeline for AAV delivery and behavioral tests. (B‐D) Mice were subjected to the Morris Water Maze test to compare the escape latency during a 5‐day training phase (B), and the number of platform region crossings (C) and the average swimming speed (D) during the probe test. (E) Mice were studied for their spontaneous alternations in the Y‐maze test. (F, G) Mice were subjected to the fear conditioning test to compare the percentage of freezing time in the cued (F) and contextual (G) phases. (H) The body weights of mice were compared. (I, J) Representative images of the hindlimb clasping (I) and the comparison of hindlimb clasping scores (J). Data are presented as mean ± SEM. *p* values were determined by one‐way analysis of variance (ANOVA) followed by Tukey's post hoc analysis in (C‐H, and J), and two‐way ANOVA followed by Tukey's post hoc analysis in (B). *n* = 9 mice per group for all these tests. ns, not significant; *p < 0.05; **p < 0.01; ***p < 0.001; ****p < 0.0001.

To investigate the therapeutic potential of astrocytic PYGM in tauopathies, we overexpressed PYGM specifically in hippocampal astrocytes via stereotaxic injection of AAV‐*gfaABC1D‐PYGM‐HA‐mCherry* into the hippocampus of PS19 mice at 6 months of age, when tau pathology has been developing but potentially still modifiable, and analyzed mouse behaviors at 9 months of age (Figure 4A and S5A). Immunofluorescence analysis confirmed predominant infection of astrocytes by AAVs expressing PYGM‐HA (Figure S5B). Immunoblotting analysis confirmed PYGM overexpression in the hippocampus of PS19 mice injected with AAVs expressing PYGM‐HA (Figure S5C, D). In the Morris Water Maze test, we found that PYGM‐overexpressing PS19 mice (PS19‐OE) had shorter escape latencies during the training phase and more platform crossings in the probe testing phase than PS19 control (PS19‐NC) mice (Figure 4B, C), though their swimming speeds were not different (Figure 4D). PS19‐OE mice also showed elevated spontaneous alternation in the Y‐maze test (Figure 4E) and enhanced cued freezing responses in the fear conditioning test (Figure 4F, G). Moreover, we found that PYGM overexpression attenuated weight loss (Figure 4H) and reduced hindlimb clasping (Figure 4I, J) in PS19 mice. For WT mice with PYGM overexpression (Figure S5E, F), we found that they showed no significant differences in performance in the Morris Water Maze, Y‐maze, and fear conditioning tests, in hindlimb clasping, or in body weight compared to WT controls (Figure 4B–J). Collectively, these results suggest that promoting astrocytic PYGM expression alleviates cognitive and motor deficits in tauopathy mice without inducing noticeable behavioral alterations under physiological conditions.

### 3.5 Astrocytic PYGM overexpression attenuates tauopathy‐related pathologies in PS19 mice

**FIGURE 5:**
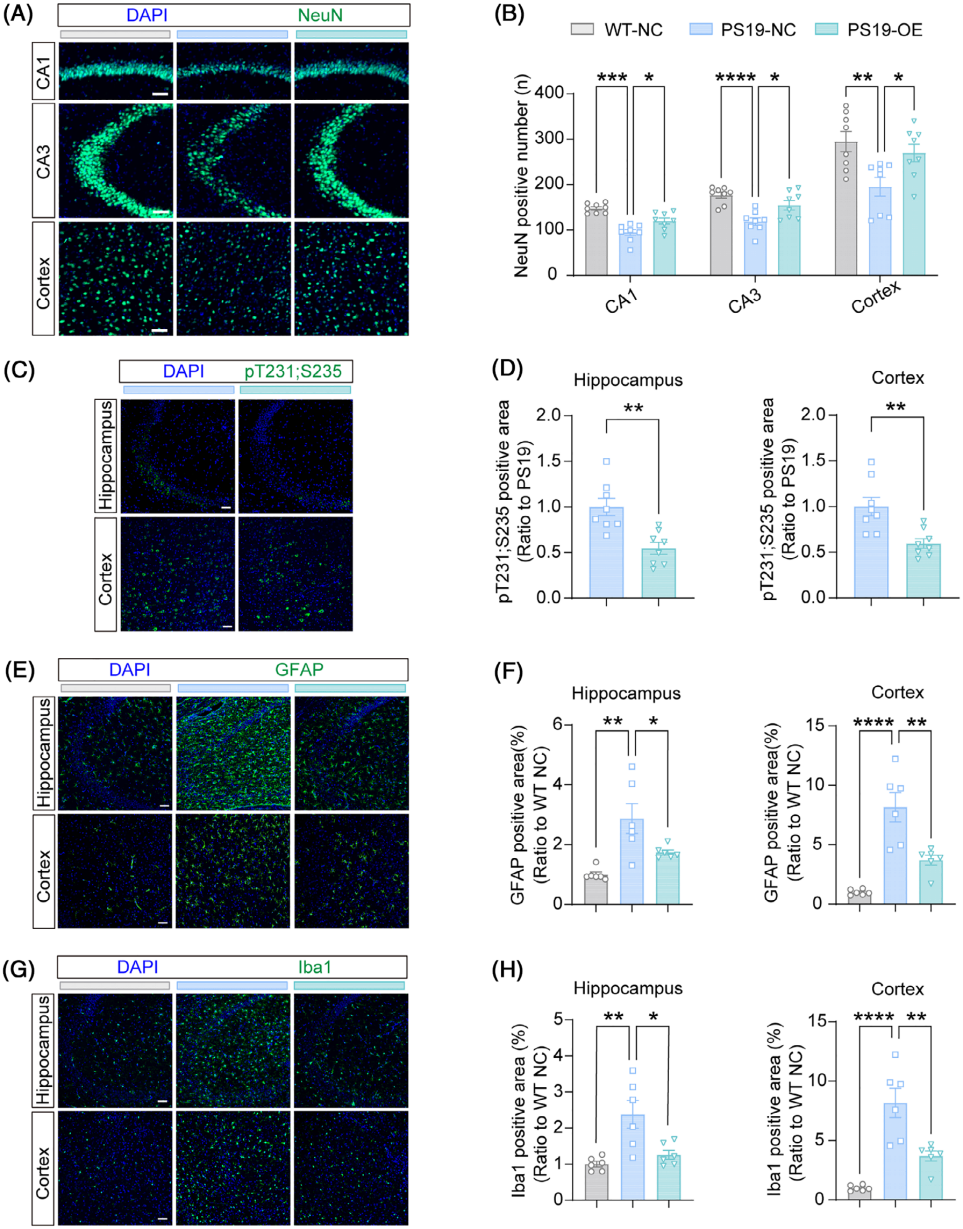
Astrocytic PYGM (glycogen phosphorylase, muscle associated) overexpression alleviates tauopathy‐related pathologies in tau^P301S^ transgenic (PS19) mice. (A, B) Immunofluorescence staining of NeuN (green) (A) and quantitative analysis (B) in brain sections from different mice. The nuclei were counterstained with 4′,6‐diamidino‐2‐phenylindole (DAPI; blue). Scale bars: 50 µm. *n* = 8 per group. (C, D) Immunofluorescence staining of phosphorylated tau at sites T231/S235 (green) (C) and quantitative analysis (D) in brain sections from different mice. The nuclei were counterstained with DAPI (blue). Scale bars: 100 µm. *n* = 8 per group. (E, F) Immunofluorescence staining of glial fibrillary acidic protein (GFAP; green) (E) and quantitative analysis (F) in brain sections from different mice. The nuclei were counterstained with DAPI (blue). Scale bars: 100 µm. *n* = 6 per group. (G, H) Immunofluorescence staining of *Iba*1 (green) (G) and quantitative analysis (H) in brain sections from different mice. The nuclei were counterstained with DAPI (blue). Scale bars: 100 µm. *n* = 6 per group. Data are presented as the mean ± SEM. *p* values were determined by one‐way analysis of variance (ANOVA) followed by Tukey's post hoc analysis in (B, F, and H), and two‐tailed unpaired Student's *t* test in (D). *p < 0.05; **p < 0.01; ***p < 0.001; ****p < 0.0001.

We also quantified NeuN‐positive cells in the hippocampal CA1 and CA3 regions and cortex in mice with astrocytic PYGM overexpression. The results showed that astrocytic PYGM overexpression alleviated neuronal loss in all these regions in the PS19 background (Figure 5A, B). TUNEL staining also showed fewer apoptotic cells in PS19‐OE mice than in PS19‐NC mice (Figure S6A, B). We further analyzed synaptic proteins and found that astrocytic PYGM overexpression attenuated the reduction of GluN2A, GluN1, GluA2, and PSD95 protein levels in PS19 mice (Figure S6C, D).

Immunofluorescence staining of phosphorylated tau at sites T231/S235 in the hippocampus and cortex showed that astrocytic PYGM overexpression reduced p‐tau expression in PS19 mice (Figure 5C, D). Consistently, immunoblot analysis revealed that astrocytic PYGM overexpression significantly reduced tau phosphorylation at multiple pathogenic sites (T212, T231/S235, and T205) without altering total tau levels in PS19 mice (Figure S6E, F).

Immunofluorescence quantification of GFAP (Figure 5E, F) and Iba1 (Figure 5G, H) showed that astrocytic PYGM overexpression reduced both of them in the hippocampus and cortex of PS19 mice, suggesting that PYGM overexpression alleviates tauopathy‐associated astrogliosis and microgliosis. Together, these results indicate that promoting astrocytic PYGM expression exerts neuroprotection in tauopathy.

### 3.6 PYGM deficiency disrupts glycogen homeostasis and energy metabolism in astrocytes

**FIGURE 6:**
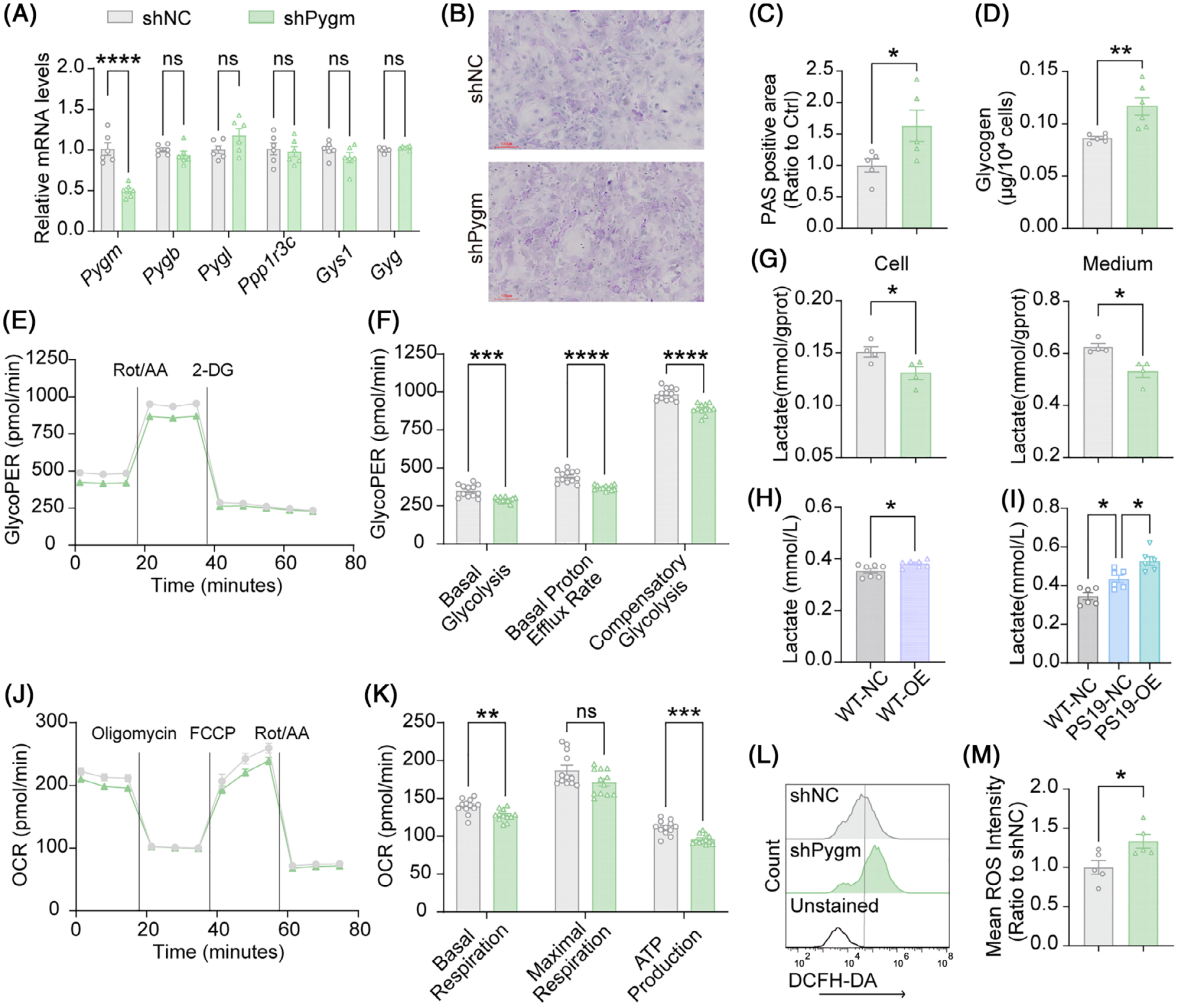
PYGM (glycogen phosphorylase, muscle associated) deficiency disrupts glycogen‐energy metabolism in astrocytes. (A) Cultured primary astrocytes from wild‐type (WT) mice were infected with lentiviruses expressing shRNA targeting *Pygm* (shPygm) or control shRNA (shNC). The mRNA levels of *Pygm*, *Pygb*, *Pygl*, *Ppp1r3c*, *Gys1*, and *Gyg* in shNC and shPygm astrocytes were analyzed by quantitative real‐time polymerase chain reaction (qRT‐PCR) for comparison. *n* = 6 experiments. (B, C) Periodic acid Schiff (PAS) staining of glycogen (magenta) in shNC and shPygm astrocytes (B) and quantitative analysis (C). Scale bars: 100 µm. *n* = 5 experiments. (D) Glycogen concentrations in shNC and shPygm astrocytes were assayed by a glycogen assay kit for comparison. *n* = 6 experiments. (E, F) Glycolysis capacity assessed by real‐time extracellular acidification rate (ECAR) monitoring in shNC and shPygm astrocytes (E) and corresponding quantitative comparisons (F). *n* = 12 experiments. (G) Lactate levels in the cell lysates and extracellular medium from shNC and shPygm astrocytes were quantified by a colorimetric assay kit for comparison. *n* = 4 experiments. (H) Comparison of lactate concentrations in the cerebrospinal fluid (CSF) of WT mice with astrocytic PYGM overexpression (WT‐OE) and their controls (WT‐NC). *n* = 7 per group. (I) Comparison of lactate concentrations in the CSF of PS19 mice with (OE) or without (NC) PYGM overexpression. *n* = 6 mice per group. (J, K) Mitochondrial respiration assessed by real‐time oxygen consumption rate (OCR) monitoring in shNC and shPygm astrocytes (J) and corresponding quantitative comparisons (K). *n* = 12 experiments. (L, M) shNC and shPygm astrocytes were stained with DCFH‐DA and analyzed by flow cytometry (L). shNC astrocytes without DCFH‐DA staining were used as a negative control. ROS levels were quantified and compared based on the mean fluorescence intensity of DCFH‐DA‐positive cells (M). *n* = 5 experiments. Data are presented as mean ± SEM. *p* values were determined by two‐tailed unpaired Student's *t* test in (A, C, D, F‐H, K, M) and one‐way analysis of variance (ANOVA) followed by Tukey's post hoc analysis in (I). ns, not significant; *p < 0.05; **p < 0.01; ***p < 0.001; ****p < 0.0001.

To elucidate the role of PYGM in astrocytic glycogen regulation, we knocked down *Pygm* expression in mouse primary astrocytes (Figure 6A). *Pygm* knockdown did not affect the mRNA levels of other key glycogen metabolic genes, including *Pygb*, *Pygl*, *Ppp1r3c*, *Gys1*, and *Gyg* (Figure 6A), but led to significantly increased intracellular glycogen accumulation (Figure 6B–D).

We next performed a comprehensive analysis of astrocytic bioenergetic function. Assessment of glycolysis capacity via ECAR demonstrated that PYGM‐deficient astrocytes exhibited marked glycolytic impairment, with significant reductions in basal glycolysis, compensatory glycolytic capacity, and basal proton efflux rate (Figure 6E, F). The defects in glycolytic metabolism were accompanied by markedly diminished lactate production, as evidenced by decreased levels of both intracellular lactate and lactate released into the extracellular medium (Figure 6G). In line with these observations, astrocyte‐specific PYGM overexpression increased lactate levels in the CSF of both WT (Figure 6H) and PS19 (Figure 6I) mice. Mitochondrial respiratory function, evaluated through OCR measurements, was similarly compromised in PYGM‐knockdown astrocytes, which displayed reduced basal respiration and impaired ATP‐linked respiration (Figure 6J, K). Moreover, PYGM knockdown dramatically elevated ROS in astrocytes (Figure 6L, M). Furthermore, PYGM knockdown promoted a pro‐inflammatory activation in astrocytes, as indicated by elevated mRNA levels of inflammatory factors *Tnfα*, *Il6*, and *Il1β* (Figure S7A–C). Collectively, these data indicate that PYGM deficiency triggers a severe bioenergetic crisis in astrocytes, characterized by compromised glycolysis, mitochondrial dysfunction, elevated oxidative stress, and enhanced neuroinflammatory responses.

### 3.7 Astrocytic PYGM deficiency impairs neuronal metabolic homeostasis and promotes neuronal tau phosphorylation

**FIGURE 7:**
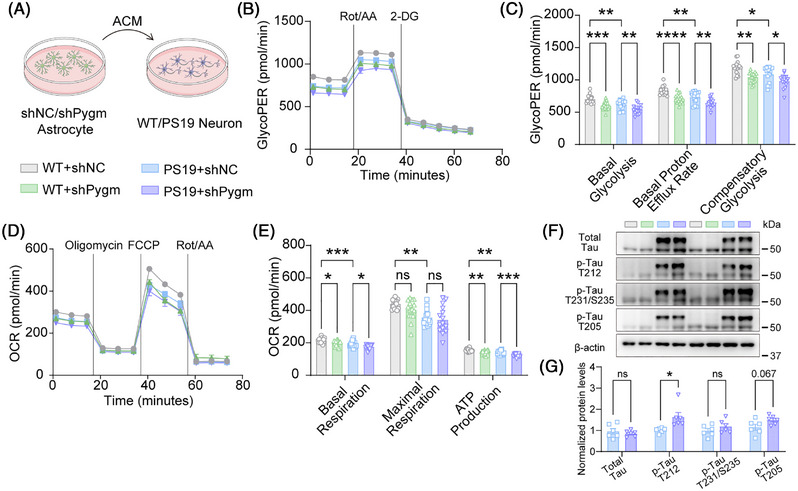
Astrocytic PYGM (glycogen phosphorylase, muscle associated) deficiency causes neuronal energetic impairment and aggravated Tau phosphorylation. (A) The conditioned media treatment scheme: conditioned media (ACM) collected from shNC or shPygm astrocytes were centrifuged at 1000 g for 10 minutes. The supernatants were used to treat wild‐type (WT) or tau^P301S^ transgenic (PS19) primary neurons for 3 days. (B, C) Glycolysis capacity measured by real‐time extracellular acidification rate (ECAR) monitoring in neurons treated with shNC or shPygm ACM (B) and corresponding quantitative comparisons (C). *n* = 16 experiments. (D, E) Mitochondrial respiration measured by real‐time oxygen consumption rate (OCR) monitoring in neurons treated with shNC or shPygm ACM (D) and corresponding quantitative comparisons (E). *n* = 15 experiments. (F, G) Immunoblot (F) and quantitative analysis (G) of total tau and phosphorylated tau at sites T212, T231/S235, and T205 in neurons treated with shNC or shPygm ACM. *n* = 6 experiments. Data are presented as mean ± SEM. *p* values were determined by two‐tailed unpaired Student's *t* test in (G), and one‐way analysis of variance (ANOVA) followed by Tukey's post hoc analysis in (C and E). ns, not significant; *p < 0.05; **p < 0.01; ***p < 0.001; ****p < 0.0001.

To further explore the downstream impact of astrocytic PYGM deficiency on neuronal function, we treated primary neurons derived from WT or PS19 mice with ACM collected from control or PYGM‐deficient astrocytes (Figure 7A). Metabolic analysis revealed that both WT and PS19 neurons incubated with ACM from PYGM‐deficient astrocytes exhibited significantly reduced glycolysis capacity (ECAR, Figure 7B, C) and impaired mitochondrial respiration (OCR, Figure 7D, E) compared to those treated with control ACM, indicating that PYGM deficiency alters the ACM composition in a manner that compromises neuronal bioenergetics. Moreover, treatment with ACM from PYGM‐deficient astrocytes significantly enhanced tau phosphorylation at T212 in PS19 neurons without affecting total tau levels (Figure 7F, G), indicating that soluble factors released by metabolically compromised astrocytes promote tau hyperphosphorylation.

Together, these findings establish a mechanistic link between astrocytic PYGM deficiency and neuronal dysfunction in tauopathy, whereby cell‐autonomous metabolic failure in astrocytes propagates to neurons through altered extracellular milieu, ultimately disrupting neuronal energy homeostasis and accelerating tau pathology.

### 3.8 Lactate supplementation attenuates multiple tauopathy‐related phenotypes in PS19 mice

**FIGURE 8:**
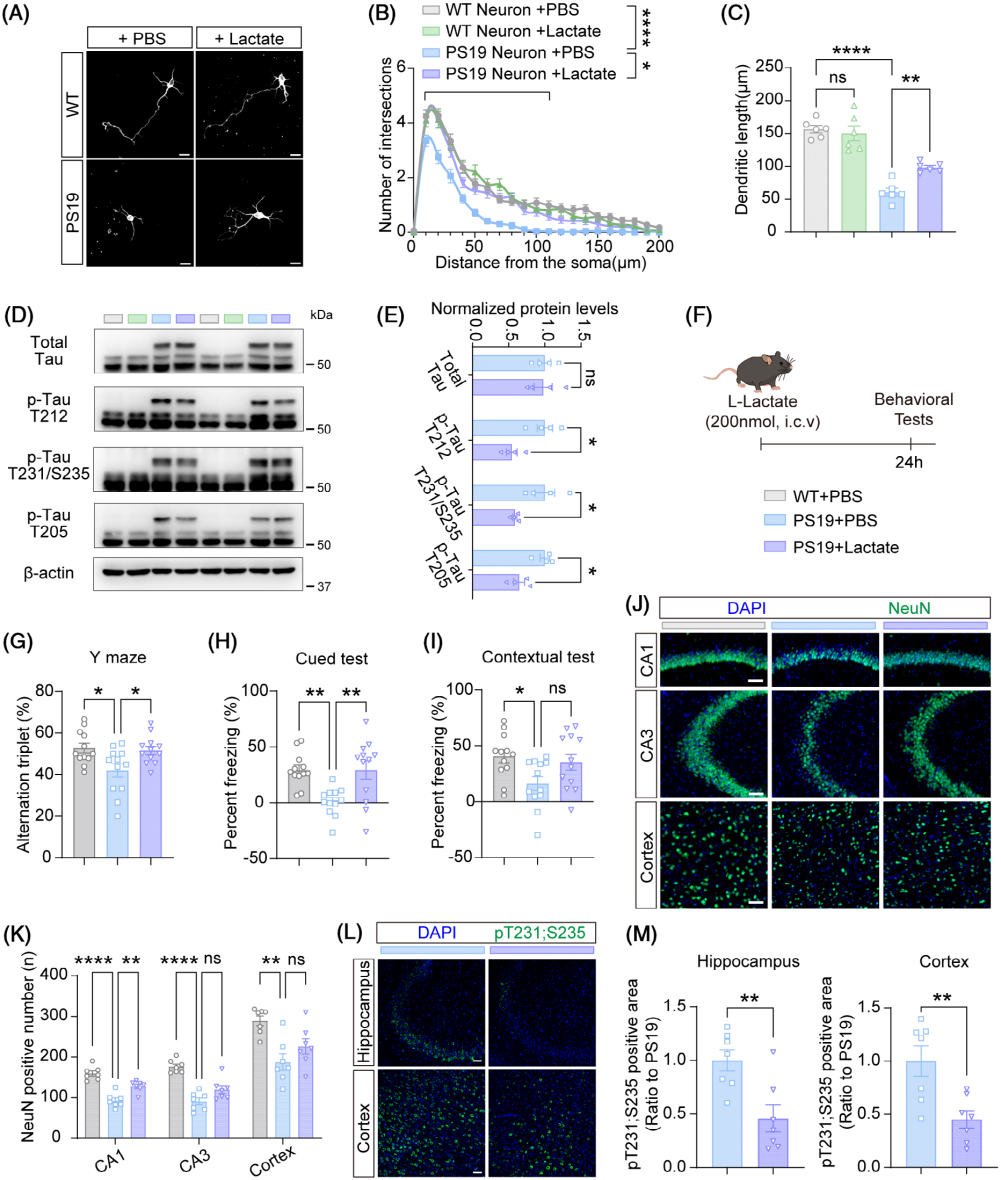
Lactate supplementation mitigates tau‐related pathologies and cognitive deficits in tau^P301S^ transgenic (PS19) mice. (A‐C) Immunofluorescence staining of Tuj1 (white) in wild‐type (WT) and PS19 primary neurons treated with PBS or lactate for 3 days (A). Scale bars: 20 µm. Quantitative comparisons of dendritic complexity using the Sholl analysis (B) and total dendritic branch length (C). *n* = 6 experiments with 50 neurons studied per group. (D, E) Immunoblot (D) and quantitative analysis (E) of total tau and phosphorylated tau at sites T212, T231/S235, and T205 in WT and PS19 neurons treated with phosphate buffered saline (PBS) or lactate. *n* = 4 experiments. (F) Schematic timeline for the in vivo L‐lactate administration and subsequent behavioral tests. (G) Mice were studied for their spontaneous alternations in the Y‐maze test. *n* = 12 mice per group. (H, I) Mice were subjected to the fear conditioning test to compare the percentage of freezing time in the cued (H) and contextual (I) phases. *n* = 12 mice per group. (J, K) Immunofluorescence staining of NeuN (green) (J) and quantitative analysis (K) in brain sections from different mice. The nuclei were counterstained with 4′,6‐diamidino‐2‐phenylindole (DAPI; blue). Scale bars: 50 µm. *n* = 7 per group. (L, M) Immunofluorescence staining of phosphorylated tau at sites T231/S235 (green) (M) and quantitative analysis (N) in brain sections from different mice. The nuclei were counterstained with DAPI (blue). Scale bars: 100 µm. *n* = 7 per group. Data are presented as mean ± SEM. *p* values were determined by one‐way analysis of variance (ANOVA) followed by Tukey's post hoc analysis in (C, G‐I, and K), two‐way ANOVA followed by Tukey's post hoc analysis in (B), and two‐tailed unpaired Student's *t* test in (E and M). ns, not significant; *p < 0.05; **p < 0.01; ****p < 0.0001.

Given that a key role of astrocytic bioenergetic activity is to produce lactate as a metabolic substrate for neurons,^36^ we hypothesized that PYGM‐regulated lactate production in astrocytes plays an important neuroprotective role against tau pathology. To test this hypothesis, we first treated PS19 neurons with L‐lactate. L‐lactate treatment significantly improved compromised dendritic complexity in PS19 neurons, as evidenced by enhanced Sholl intersections and increased total dendritic branch length (Figure 8A–C). L‐lactate treatment also reduced phosphorylated tau levels without affecting total tau in PS19 neurons (Figure 8D, E). Importantly, this effect appears to be specific to L‐lactate, as treatments with glucose and oleic acid had no effect on reducing tau phosphorylation in PS19 neurons (Figure S8A–D).

We further evaluated the therapeutic potential of lactate administration in vivo by intracerebroventricular injection of L‐lactate in 9‐month‐old PS19 mice (Figure 8F). Behavioral assessments conducted 24 hours post‐injection revealed that L‐lactate treatment rescued spontaneous alternation deficiency in PS19 mice in the Y‐maze test (Figure 8G) and reversed decreased freezing percentage in PS19 mice in the cued fear conditioning test (Figure 8H, I). Quantification of NeuN‐positive cells revealed that L‐lactate treatment increased neuronal numbers in the CA1 region of PS19 mice (Figure 8J, K). Consistently, L‐lactate treatment reduced apoptotic cell death as indicated by TUNEL staining (Figure S8E, F).

Immunoblot analysis showed that L‐lactate treatment elevated protein levels of GluN2A, GluN2B, GluA1, GluA2, PSD95, and PSD93 (Figure S8G, H). In addition, L‐lactate treatment reduced tau phosphorylation at sites T212 and T231/S235 in PS19 mice (Figure 8L–M and S8I–J). Moreover, L‐lactate treatment attenuated astrogliosis and microgliosis, as shown by decreased GFAP and IBA1 immunoreactivities in the hippocampus and cortex (Figure S8K–N )。

These results demonstrate that lactate supplementation effectively mitigates synaptic protein deficits, reduces tau hyperphosphorylation, suppresses gliosis, and decreases apoptosis in PS19 mice, supporting the therapeutic potential of targeting lactate metabolism in tauopathy.

## 4 DISCUSSION

Tauopathies are neurodegenerative disorders characterized by the abnormal accumulation of hyperphosphorylated tau, leading to synaptic failure, gliosis, neuronal loss, and cognitive and/or motor deficits.^1^, ^3^ Glycogen in astrocytes serves as the primary glucose reserve in the brain and plays a crucial role in supporting neuronal function.^8^, ^36^ Recent studies show that astrocytes modulate disease progression by mobilizing their glycogen stores to fuel neurons under metabolic stress.^37^, ^38^, ^39^ Upregulated astrocyte glycolysis and glycogenolysis have been shown to improve cognition, support neuronal survival, and promote axonal growth in the 5×FAD Alzheimer's model.^40^ Conversely, impaired astrocytic glycolysis increases the accumulation of amyloid‐β (Aβ) within and around astrocytes, thereby heightening their vulnerability to Aβ toxicity.^41^

On the other hand, glycogen metabolism also occurs in neurons, and dysregulation of neuronal glycogen metabolism similarly impairs neuronal functions and animal behaviors and induces neurodegenerative phenotypes. For example, abnormally synthesized glycogen in neurons leads to neuronal apoptosis.^16^ PYGM knockdown in neurons results in impaired synaptic plasticity and memory in mice.^19^ One recent study shows that tau binds glycogen and their interaction forms a vicious cycle to exacerbate pathologies in tauopathy, whereas breaking down neuronal glycogen ameliorates disease‐related phenotypes in tauopathy models.^22^ Together, these findings suggest that glycogen metabolism homeostasis plays a crucial neuroprotective role in tauopathy.

In this context, we studied and observed a significant increase in the glycogenolytic rate‐limiting enzyme PYGM in astrocytes of PS19 mice at pathological stages. PYGM encodes the muscle isoform of glycogen phosphorylase, initially known for its role in glycogen catabolism and its association with McArdle disease, a metabolic myopathy caused by PYGM deficiency.^42^, ^43^ Although typically expressed in skeletal muscle, PYGM also presents in the brain.^44^ Importantly, a similar elevation of PYGM expression was observed in human FTLD‐U brain samples, reinforcing the clinical relevance of this astrocytic metabolic adaptation in tauopathy. However, the increase in PYGM in PS19 mice and FTLD‐U patients is surprising, as we previously found that PYGM protein levels were decreased in the brains of AD patients and APP/PS1 mice that exhibit Aβ pathology.^19^ This divergent pattern may reflect a fundamental difference in how Aβ and tau pathologies affect the metabolic environment in the brain. Compelling evidence from human neuroimaging indicates that tau pathology, rather than Aβ, is directly and dominantly linked to progressive cerebral hypometabolism in most brain regions.^45^, ^46^, ^47^ This implies that tau pathology may be more important than Aβ in driving energy depletion, leading to a severe neuronal energy crisis. Since the marked upregulation of PYGM occurs at relatively late pathological stages of tauopathy, we propose that the upregulation of astrocytic PYGM in tauopathies represents a crucial compensatory metabolic response, an attempt to bolster glycogen‐derived energy support to counteract tau‐induced energy deficits. Conversely, the Aβ‐dominant environment may engage a different set of molecular signals, potentially involving chronic neuroinflammation or distinct cellular stress responses that ultimately suppress this glycogenolytic rescue pathway, leading to PYGM downregulation.^48^

Our previous research suggests that neuronal PYGM supports synaptic activity and memory function in AD by enhancing energy mobilization.^19^ This raises a critical question: does the upregulation of astrocytic PYGM in primary tauopathy conditions represent a harmful shift contributing to pathology, or a protective, compensatory mechanism aimed at supporting energy homeostasis under metabolic stress? To answer this question, we developed an AcKO mouse model. Loss of PYGM in astrocytes led to marked impairments in synaptic plasticity, dendritic spine density, and learning and memory. Moreover, the incorporation of AcKO into the PS19 mouse model significantly exacerbated cognitive and motor deficits, body weight loss, synaptic protein depletion, gliosis, tau hyperphosphorylation, and neuronal loss. Because the *Aldh1l1*‐Cre^ER^ system we used to generate AcKO also targets peripheral immune cells during neuroinflammation,^49^ it is possible that some phenotypes observed are due to peripheral loss of PYGM. However, since specific overexpression of PYGM in astrocytes in the hippocampus substantially improved disease‐related phenotypes in PS19 mice, we believe that the disease‐modifying mechanism exerted by PYGM is primarily CNS‐autonomous.

Furthermore, we found that astrocytic PYGM deficiency resulted in glycogen accumulation, glycolysis impairment, mitochondrial respiration reduction, ATP depletion, increased ROS and pro‐inflammatory factor generation, and diminished lactate production, collectively leading to widespread energy deficits in astrocytes. When both WT and PS19 neurons were exposed to conditioned medium from PYGM‐deficient astrocytes, they exhibited impaired energy metabolism and enhanced tau phosphorylation. On the other hand, astrocytic PYGM overexpression restricted to the hippocampus markedly attenuated disease‐related pathologies not only in the hippocampus but also in the cortex. These results underscore that astrocytic PYGM deficiency not only disrupts glycogen‐energy metabolism autonomously, but also deprives neurons of essential energy support and promotes neurotoxicity, possibly through paracrine pathways.

Herein, we found that astrocytic PYGM overexpression had no adverse effects on WT mice. Several other studies also suggest that astrocytes exhibit significant metabolic plasticity, and enhancing their glycolytic output under pathological conditions may augment neuroprotective capacity rather than cause dysfunction. For example, GLUT1 deletion in astrocytes enhanced glucose metabolism and resilience to stroke.^50^ Overexpression of glycogen phosphorylase in astrocytes reduced ROS and boosted antioxidants by fueling the pentose phosphate pathway.^51^ Nevertheless, our study, as well as others, was conducted over a relatively short period (up to 3 months). Whether longer‐term overactivation of astrocytic metabolism leads to unintended consequences deserves further scrutiny.

Lactate, once considered merely a metabolic byproduct, is now recognized as a key energy substrate and signaling molecule in the brain.^52^, ^53^ Inhibiting glycogen breakdown or astrocytic lactate transport impairs memory and LTP in the hippocampus, while lactate but not glucose can rescue these deficits, suggesting energy‐independent roles in synaptic plasticity.^17^, ^54^ Lactate is taken up by neurons via monocarboxylate transporter‐2 (MCT‐2) and promotes the expression of the activity‐regulated cytoskeletal‐associated protein (Arc), phosphorylation of cAMP response element‐binding protein (p‐CREB), and phosphorylation of cofilin, all of which contribute to memory consolidation.^54^, ^55^, ^56^, ^57^ These lactate‐driven processes likely support synaptic changes underlying long‐term memory, though the mechanisms remain largely unclear. Here, we found that PYGM deficiency in astrocytes reduced lactate production, whereas PYGM overexpression increased lactate levels in mouse CSF. These findings raise the possibility that astrocytic PYGM deficiency impairs neuronal health by reducing lactate supply. Indeed, supplementation with exogenous lactate, but not glucose or oleic acid, in vitro improved dendritic morphology defects and reduced tau phosphorylation in tauopathy neurons. More importantly, intracerebroventricular administration of lactate effectively ameliorated tau pathologies and related behavioral deficits in PS19 mice. These findings suggest that lactate acts as a key downstream mediator of the protective effects of astrocytic PYGM. Nevertheless, since PYGM regulates astrocytic energy metabolism globally, including ROS and pro‐inflammatory factor generation, altered downstream products resulting from PYGM overexpression may also contribute to neuroprotection.

In summary, our findings highlight astrocytic PYGM as a crucial regulator of brain energy homeostasis and a key determinant of neuronal survival in tauopathies. Enhancing PYGM activity or lactate delivery may represent a novel therapeutic strategy to restore astrocyte‐neuron metabolic coupling.

The present study has several limitations. First, only male mice were studied. Since sex differences affect tau aggregation and neurodegeneration, it will be essential to determine whether the identified mechanisms operate similarly in females and to examine potential sex‐dependent differences in astrocytic metabolic responses to tauopathy. Second, the signals that induce the compensatory upregulation of PYGM in astrocytes remain unknown. We found that PYGM upregulation occurred at a relatively late pathological stage, suggesting that accumulated tau pathology triggers PYGM change. However, whether and how tau pathologies in neurons induce astrocytic PYGM upregulation has yet to be determined. Third, while our data clearly establish lactate as a crucial downstream effector underlying astrocytic PYGM‐mediated neuroprotection, whether and how other altered downstream products resulting from astrocytic PYGM overexpression contribute to the neuroprotection remains to be investigated.

## CONFLICT OF INTEREST STATEMENT

The authors have declared that no conflict of interest exists. Author disclosures are available in the supporting information

## CONSENT STATEMENT

Consent was not necessary for the authors.

## Supporting information



Supporting information

Supporting information

Supporting information

Supporting information

## Data Availability

All data are available upon reasonable request.
