# Supplementary material for: Astrocytic PYGM attenuates tau pathology by promoting lactate‐mediated neuroprotection
Source: Alzheimers Dement. 2026 Feb 17;22(2):e71202. doi: 10.1002/alz.71202 (PMC12910249; doi:10.1002/alz.71202)

Astrocytic PYGM attenuates tau pathology by promoting lactate-mediated neuroprotection

Jing Cao et al.

Supplemental Figure S1

Figure S1. Uncropped Original Immunoblot Images.

Figure 1A

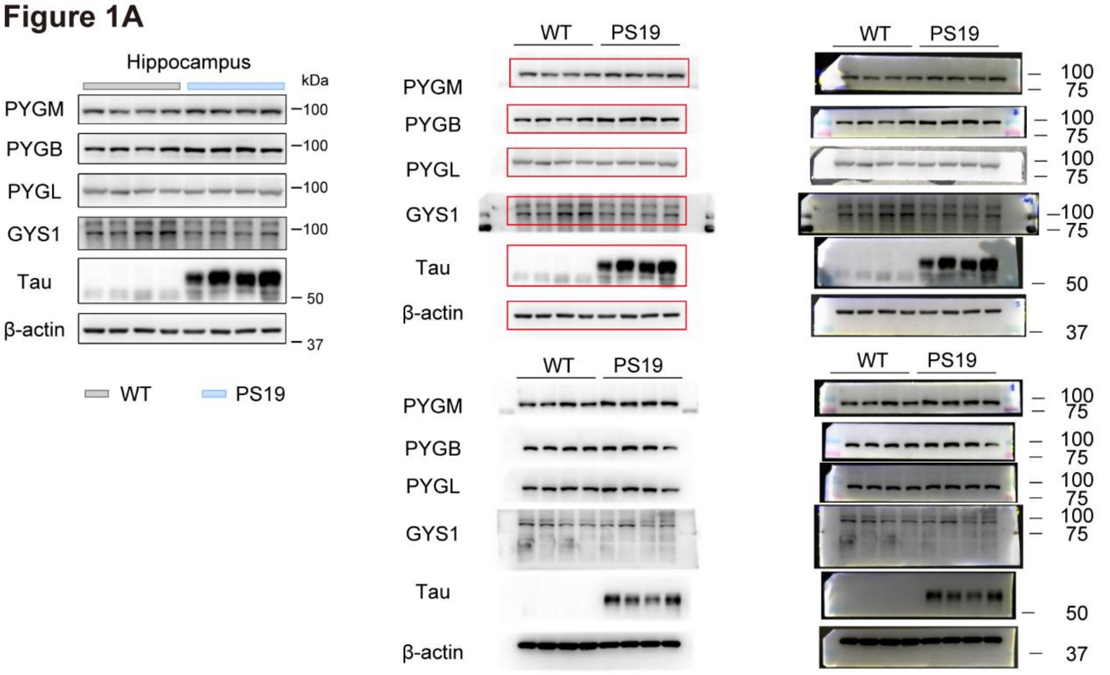

Figure 1A

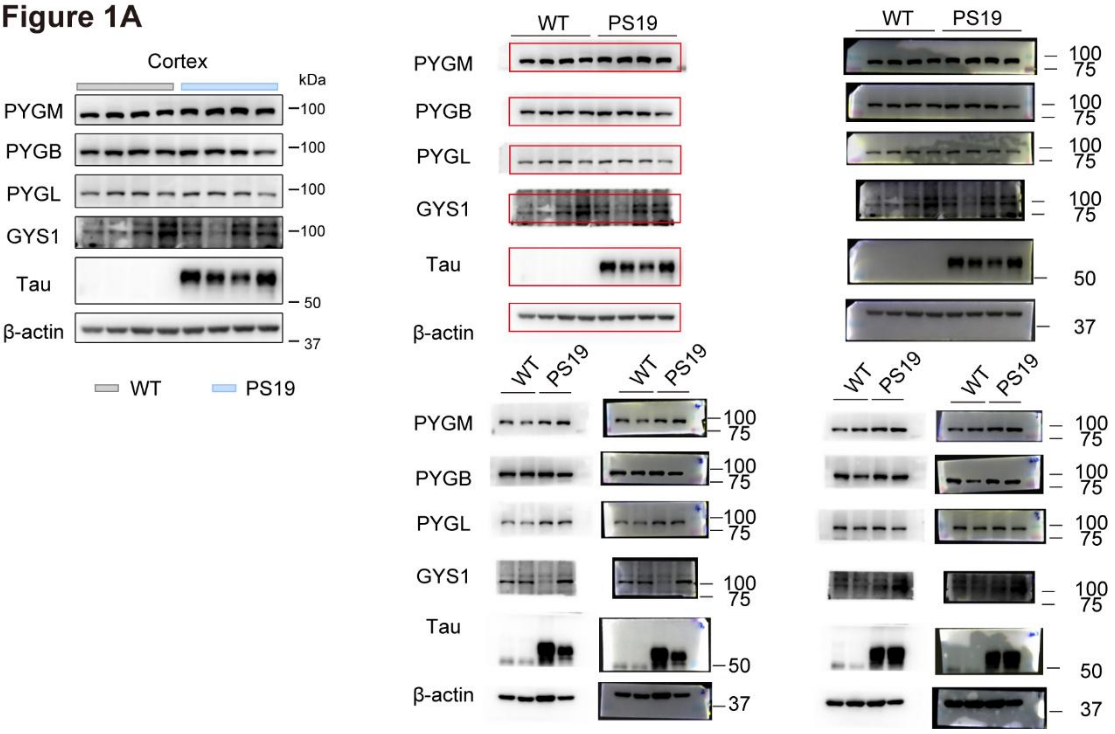

Figure 1C

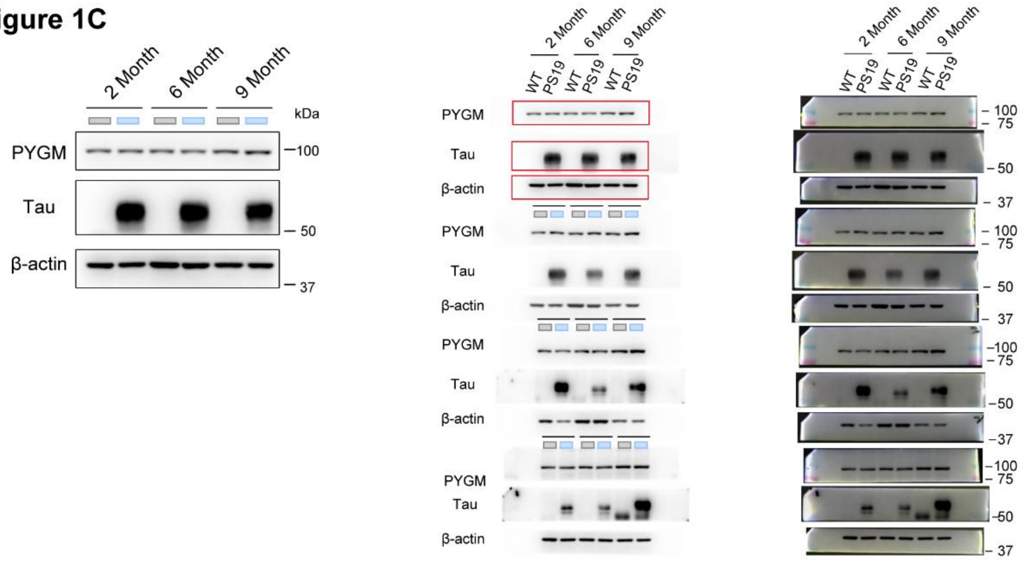

Figure 7F

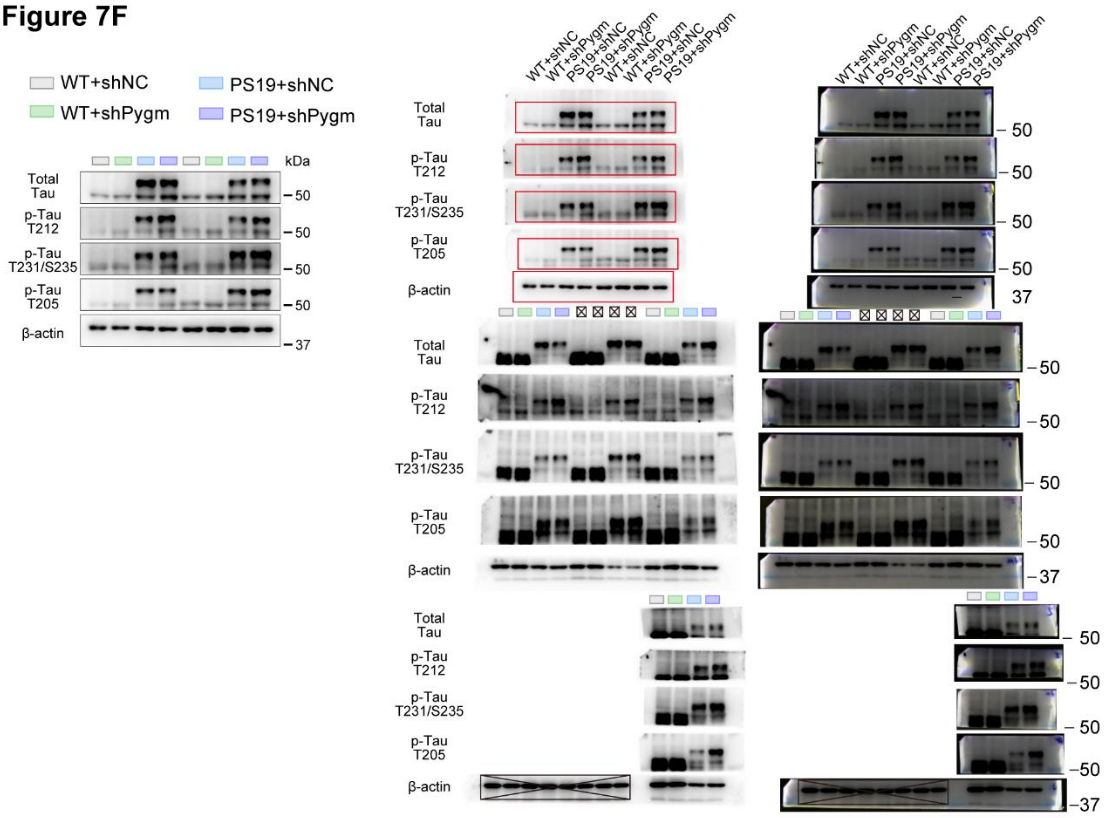

Figure 8D

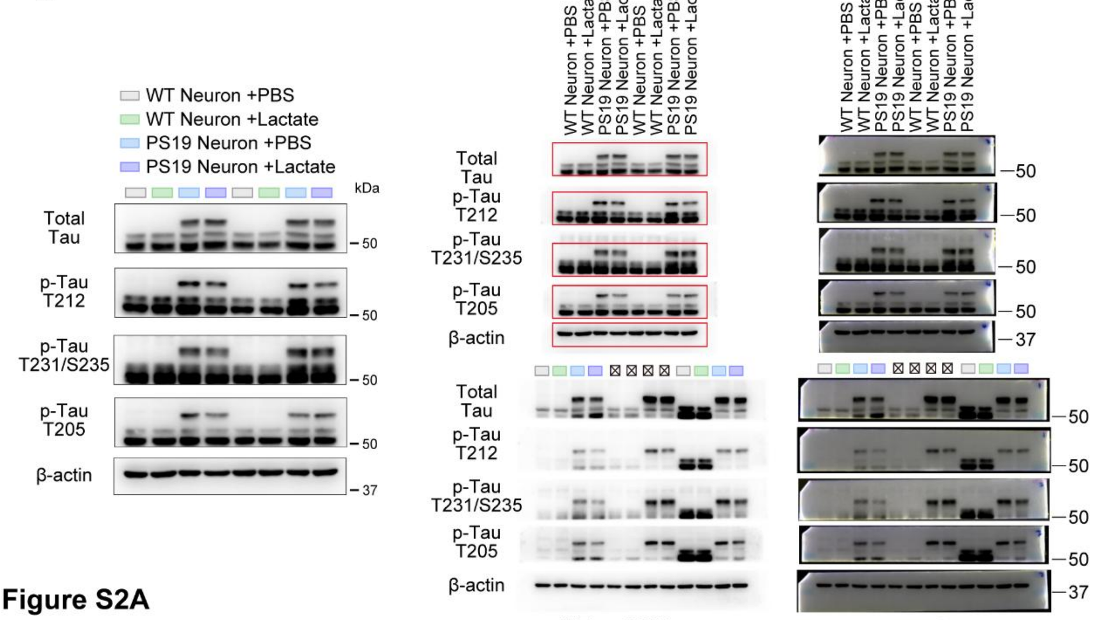

Figure S2A

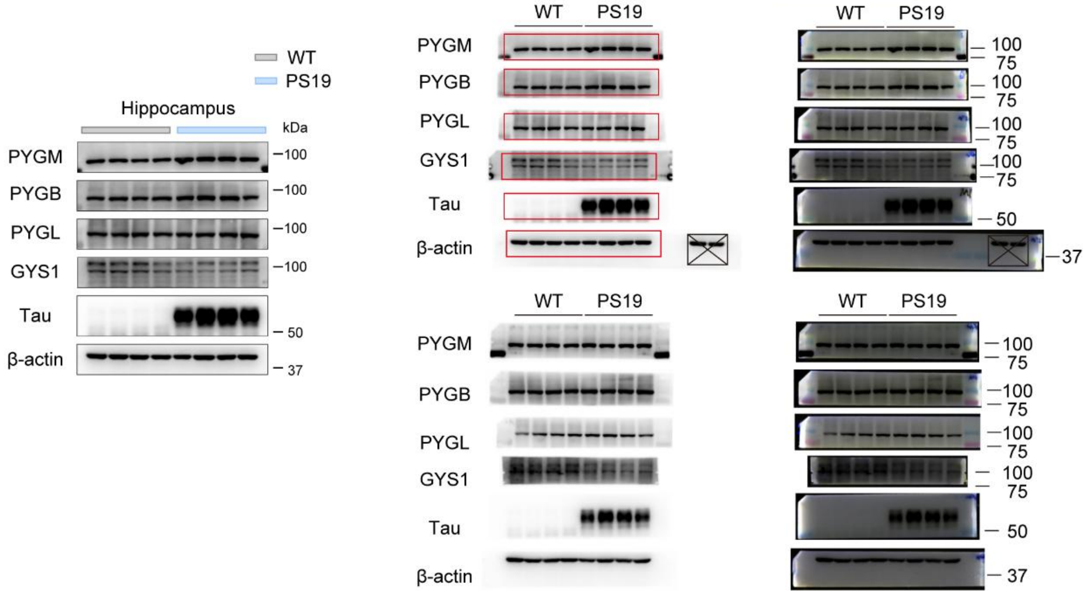

Figure S2A

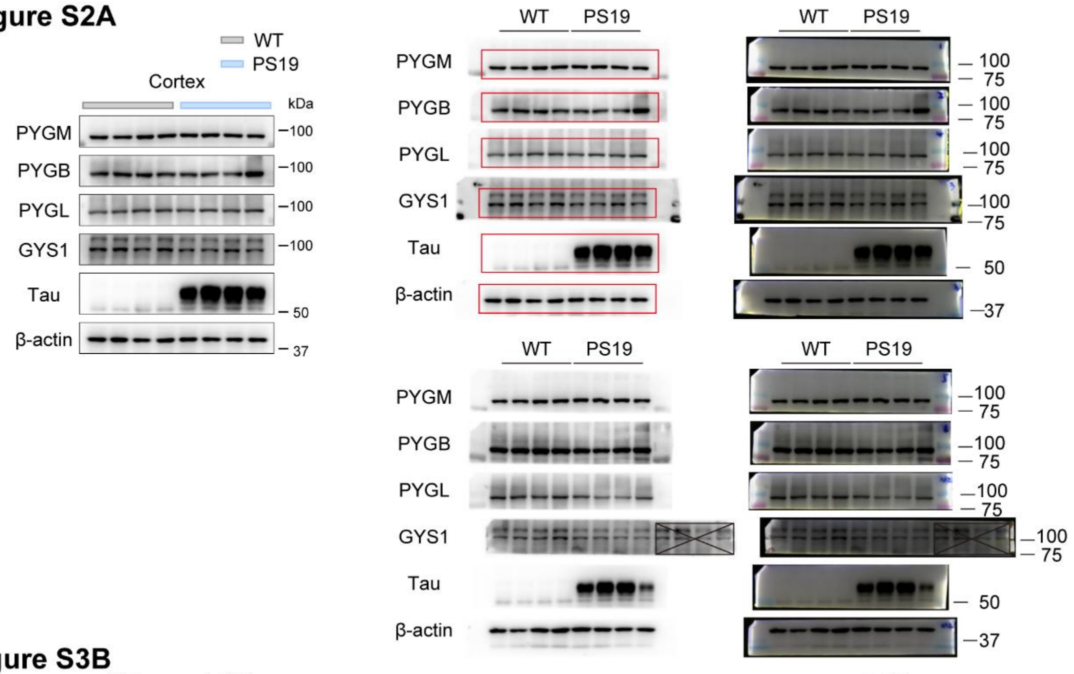

Figure S3B

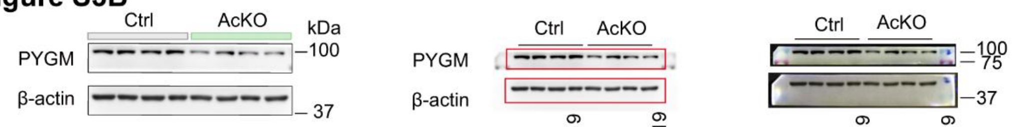

Figure S4K

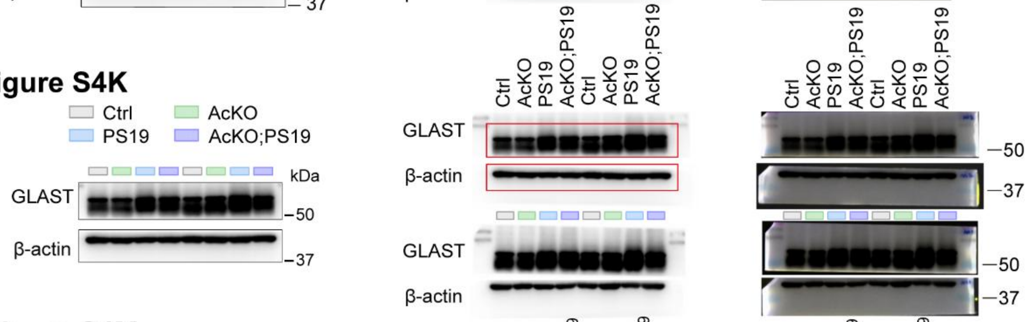

Figure S4M

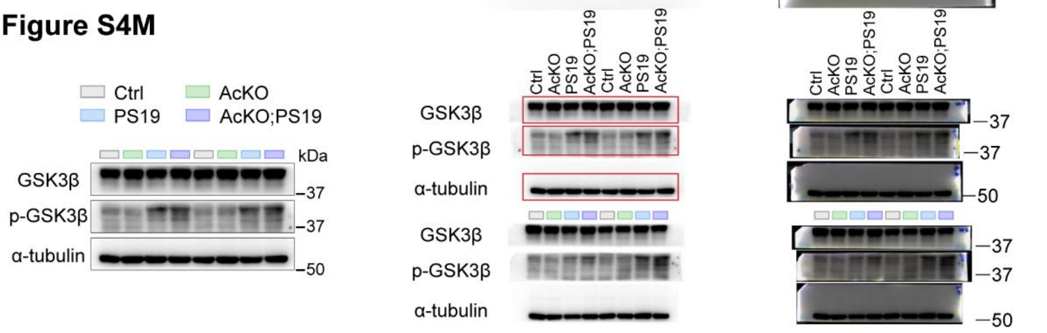

Figure S4C

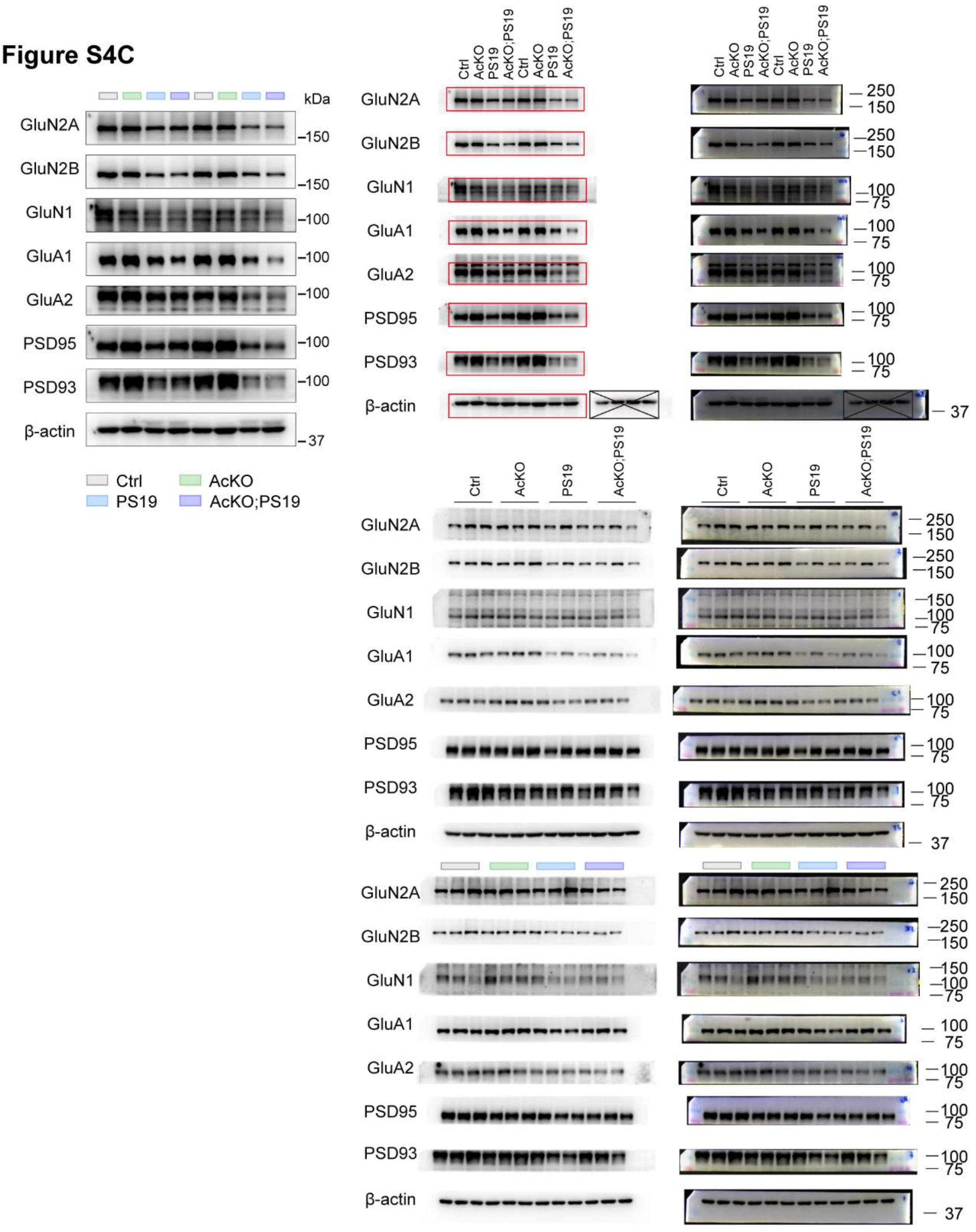
kDa
GluN2A
GluN2B
GluN1
GluA1
GluA2
PSD95
PSD93
β-actin
Ctrl
AcKO
PS19
AcKO;PS19
250
150
100
75
37

Figure S4I

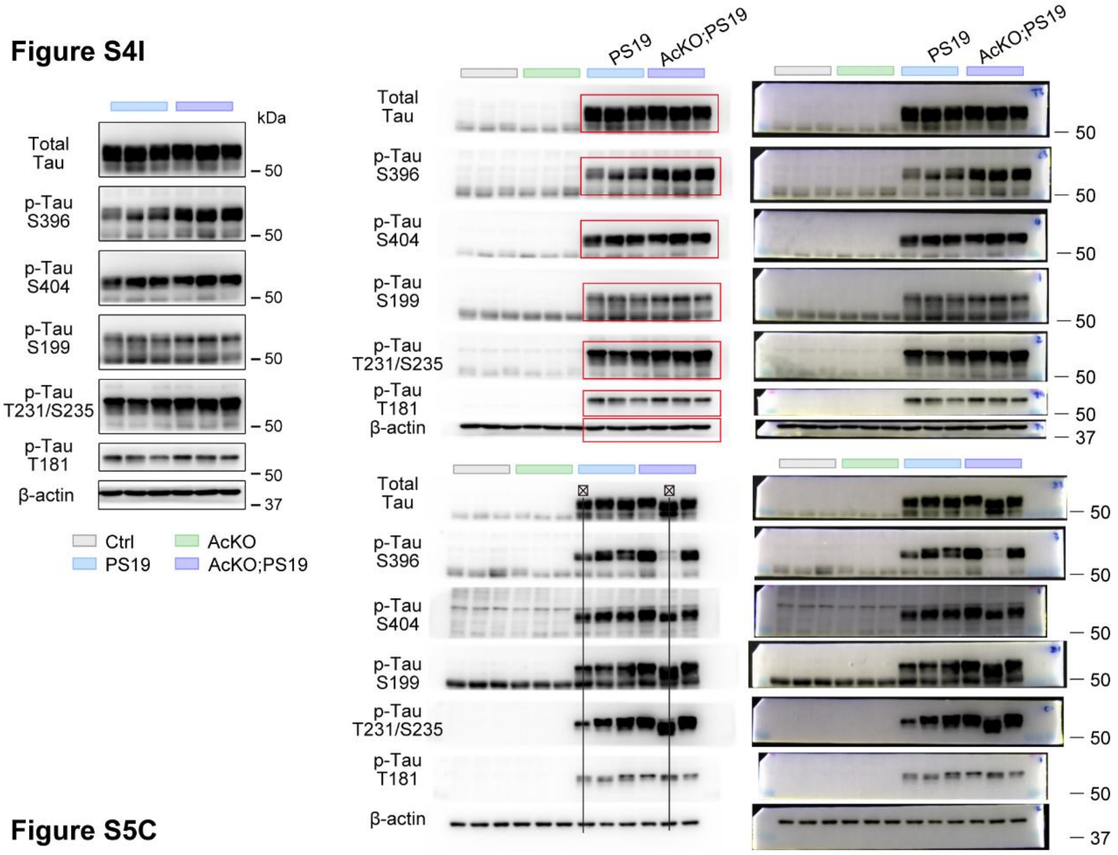

Figure S5C

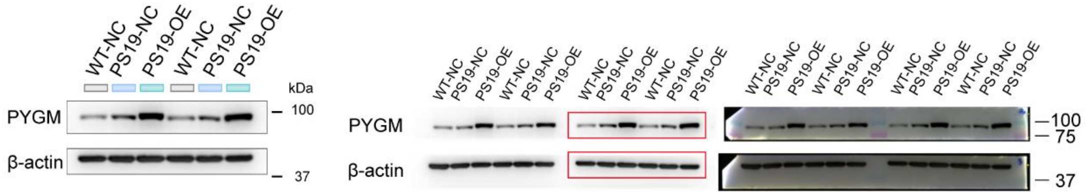

Figure S5E

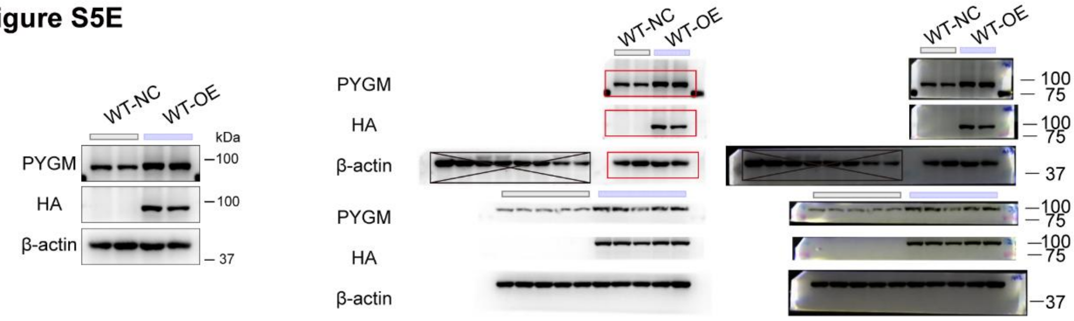

Figure S6C

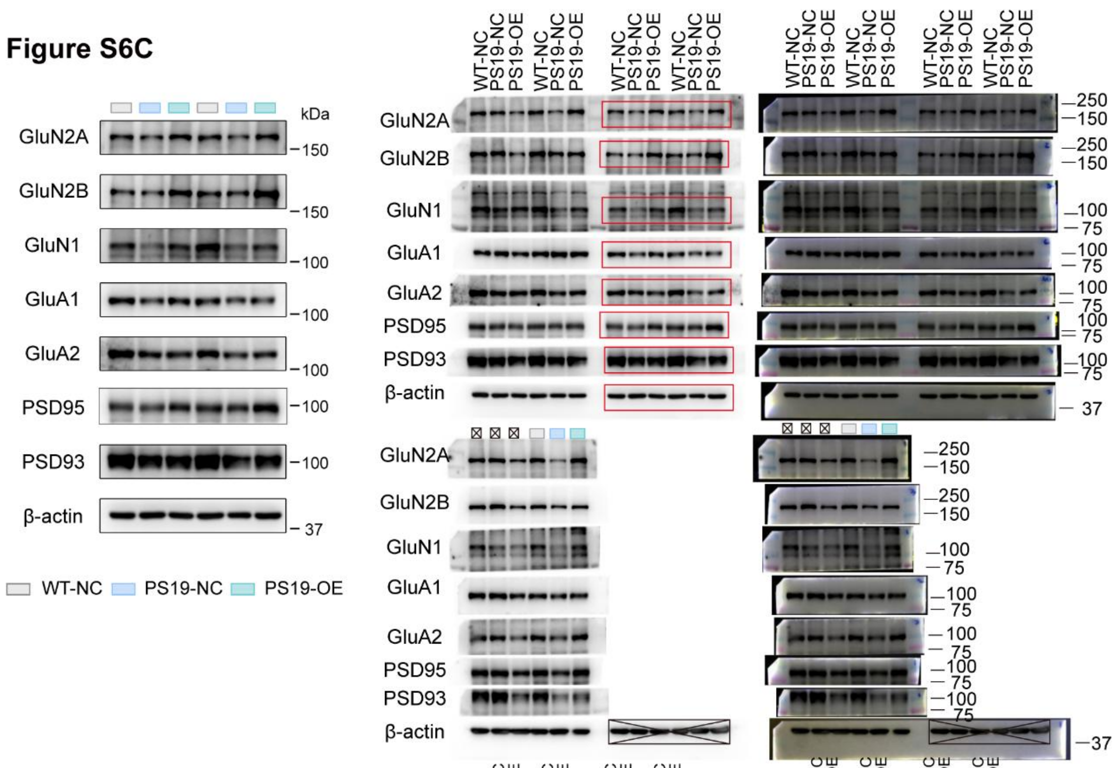

Figure S6E

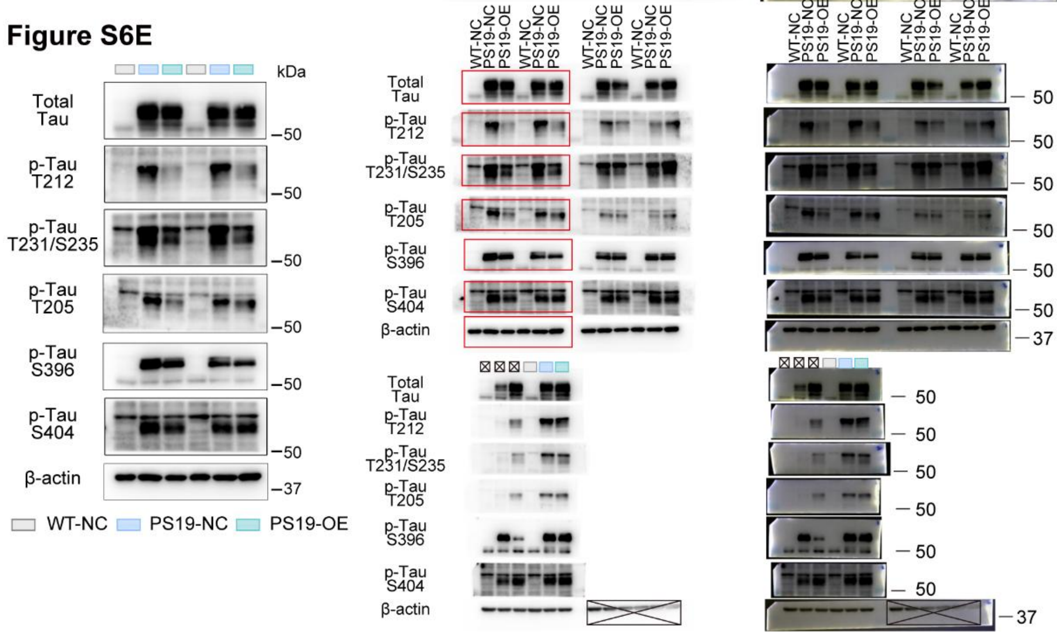

Figure S8A

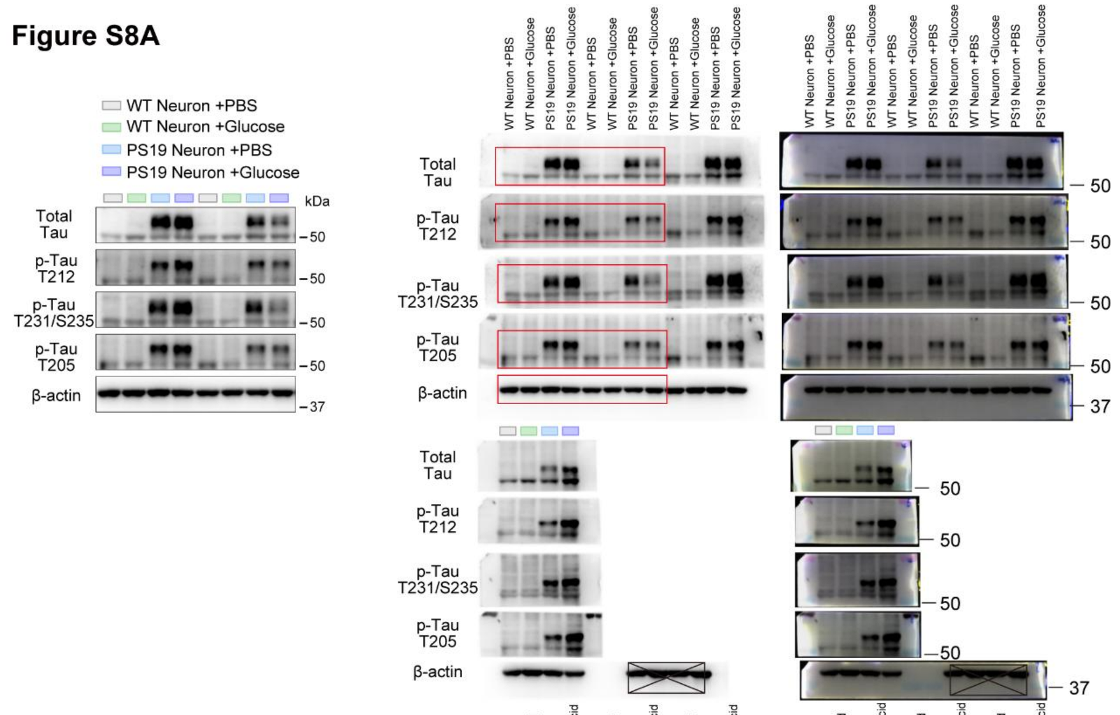

Figure S8C

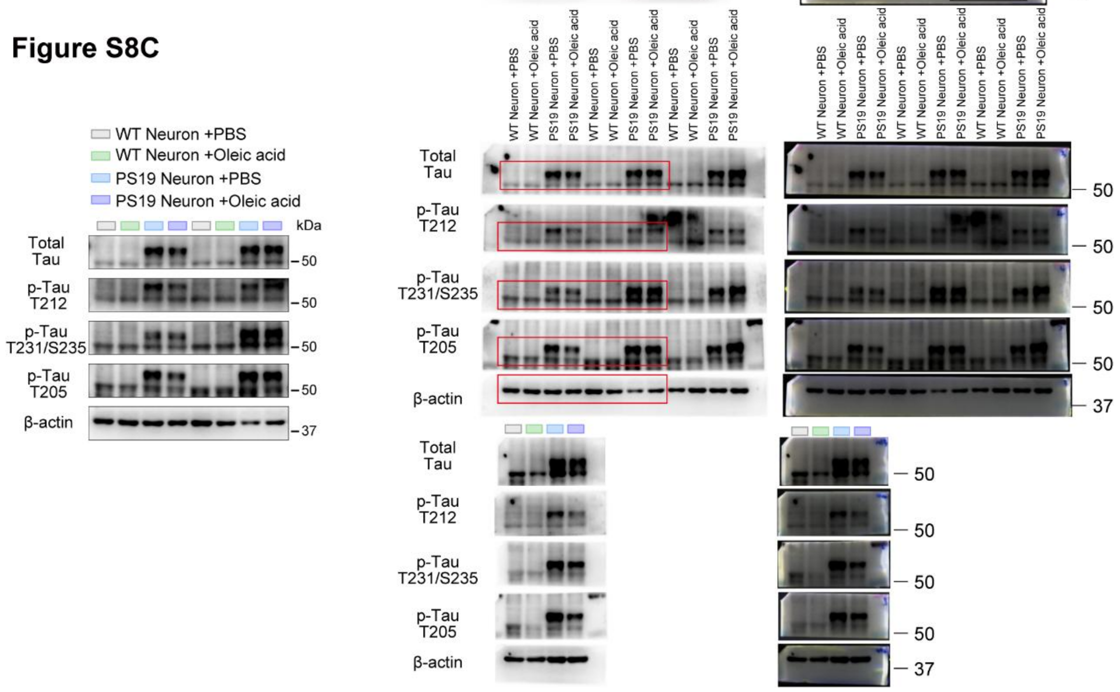

Figure S8G

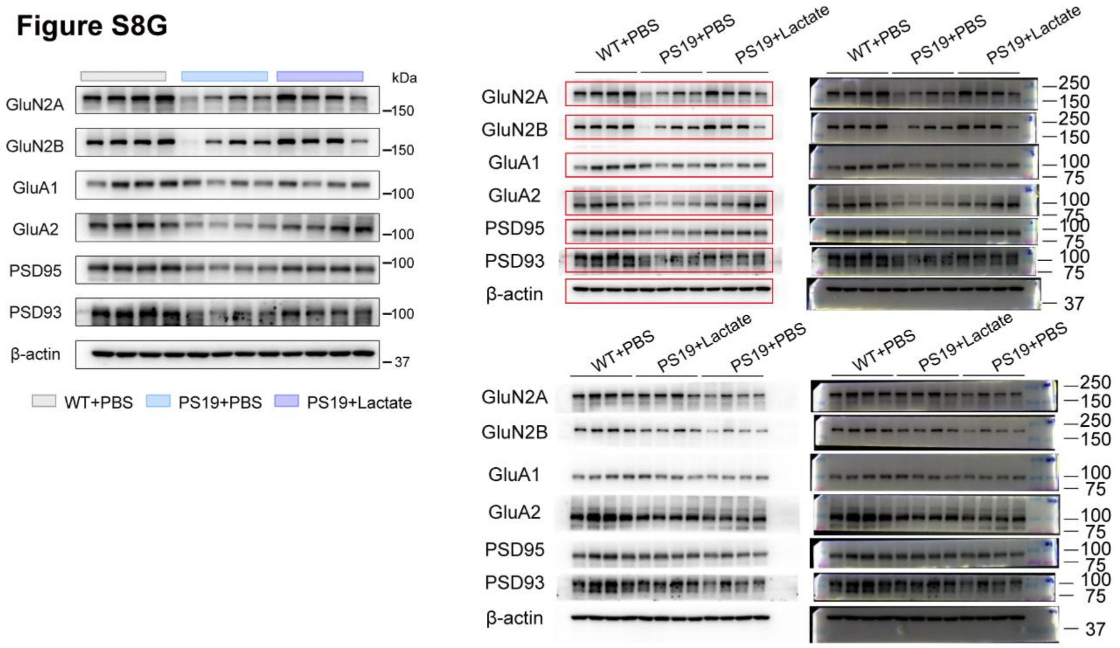

Figure S8I

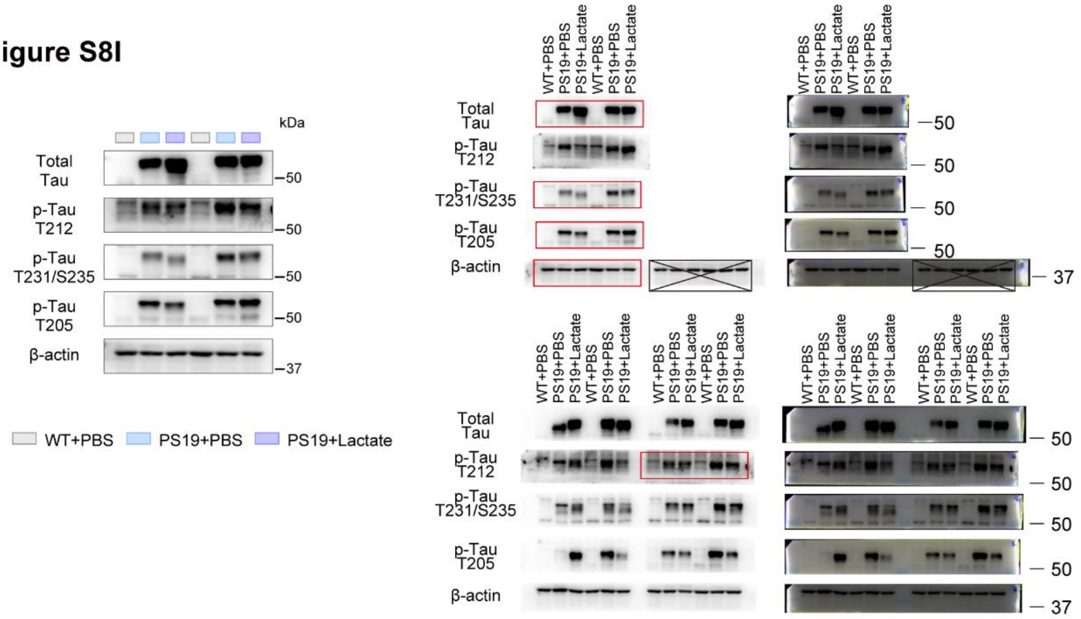

Supplement: Supplementary file 1 — Supporting information [file ALZ-22-e71202-s001.pdf]
