# Supplementary material for: Astrocytic PYGM attenuates tau pathology by promoting lactate‐mediated neuroprotection
Source: Alzheimers Dement. 2026 Feb 17;22(2):e71202. doi: 10.1002/alz.71202 (PMC12910249; doi:10.1002/alz.71202)
Supplement: Supplementary file 2 — Supporting information [file ALZ-22-e71202-s003.pdf]

Astrocytic PYGM attenuates tau pathology by promoting lactate-mediated neuroprotection

Jing Cao et al.

Supplemental Figures S2-S8.

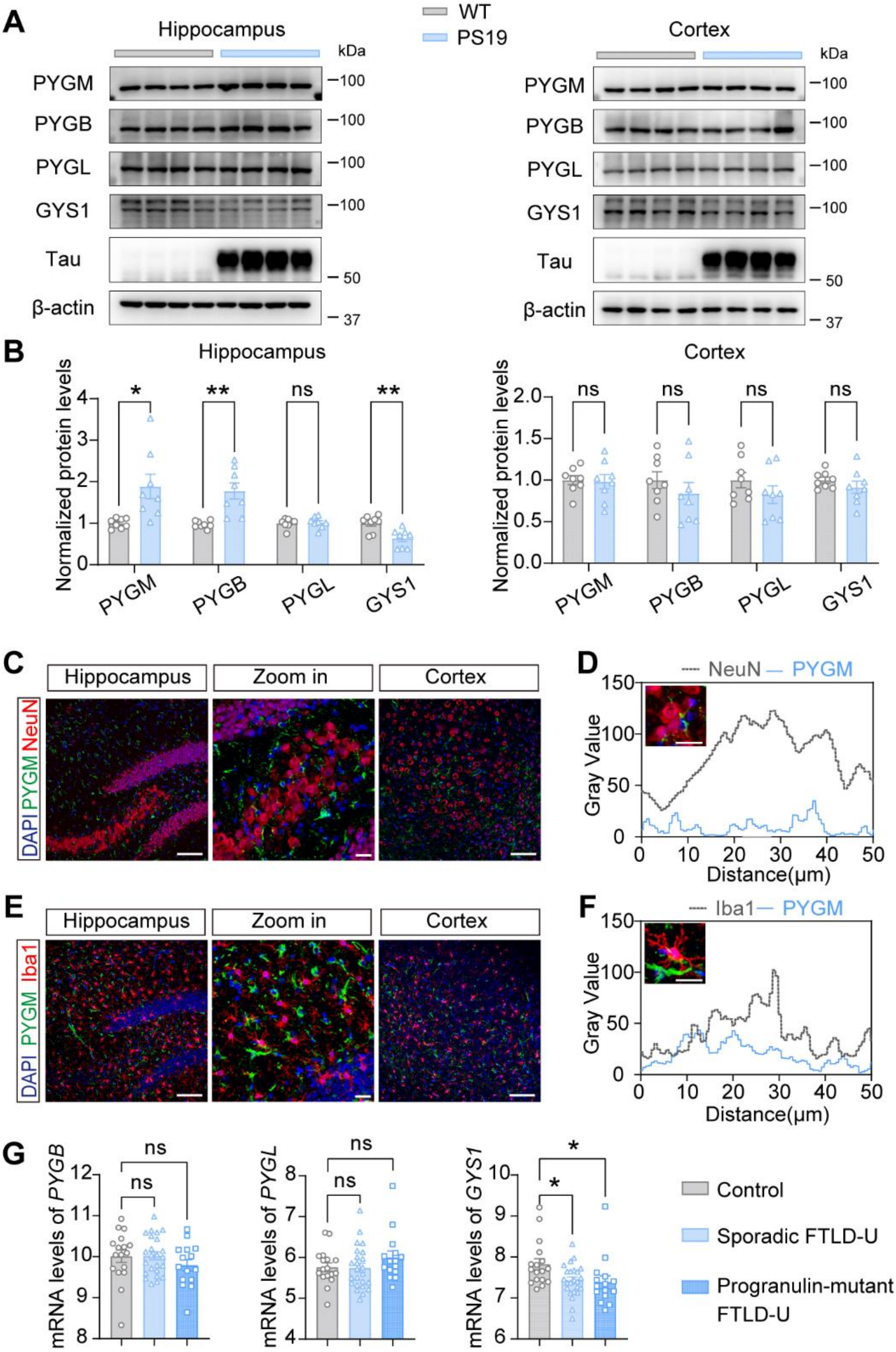

A
Hippocampus
Cortex
WT
PS19
kDa
PYGM
PYGB
PYGL
GYS1
Tau
β-actin
100
50
37
B
Normalized protein levels
ns
*
**
C
Hippocampus
Zoom in
Cortex
DAPI PYGM NeuN
D
NeuN — PYGM
Gray Value
Distance(μm)
E
DAPI PYGM Iba1
F
Iba1— PYGM
G
mRNA levels of PYGB
mRNA levels of PYGL
mRNA levels of GYS1
Control
Sporadic FTLD-U
Progranulin-mutant FTLD-U

Figure S2. PYGM and related protein expression in the brains of PS19 mice and FTLD-U patients. (A, B) Immunoblot (A) and quantitative analysis (B) of PYGM, PYGB, PYGL, and GYS1 protein levels in the hippocampus and cortex of 9-month-old female PS19 mice and WT littermates. n = 8 mice per group. (C) Representative immunofluorescence images of PYGM (green) and the neuronal marker NeuN (red) in the hippocampus and cortex of 9-month-old PS19 mice. The nuclei were counterstained with DAPI (blue). Scale bars: 100 μm for regular and 20 μm for hippocampus zoom-in. (D) Line scan analysis of PYGM and NeuN colocalization. The graph shows fluorescence intensity profiles of PYGM (blue) and NeuN (gray) along the distance of the rectangular region in the inset, indicating their spatial relationship. Scale bar: 20 μm. (E) Representative immunofluorescence images of PYGM (green) and the microglial marker Iba1 (red) in the hippocampus and cortex of 9-month-old PS19 mice. The nuclei were counterstained with DAPI (blue). Scale bars: 100 μm for regular and 20 μm for hippocampus zoom-in. (F) Line scan analysis of PYGM and Iba1 colocalization. The graph shows fluorescence intensity profiles of PYGM (blue) and Iba1 (gray) along the distance of the rectangular region in the inset, indicating their spatial relationship. Scale bar: 20 μm. (G) Comparison of *PYGB*, *PYGL*, and *GYS1* mRNA levels in post-mortem brain tissues of FTLD-U patients with progranulin gene mutations (progranulin-mutant FTLD-U, n = 15), FTLD-U patients without progranulin gene mutations (sporadic FTLD-U, n = 24), and healthy controls (control, n = 17) from the GSE13162 dataset**.** Data are presented as mean ± SEM. *P* values were determined by two-tailed unpaired Student's *t* test in (B and F), and one-way ANOVA followed by Tukey's post

hoc analysis in (G). ns, not significant; *$P < 0.05$; **$P < 0.01$.

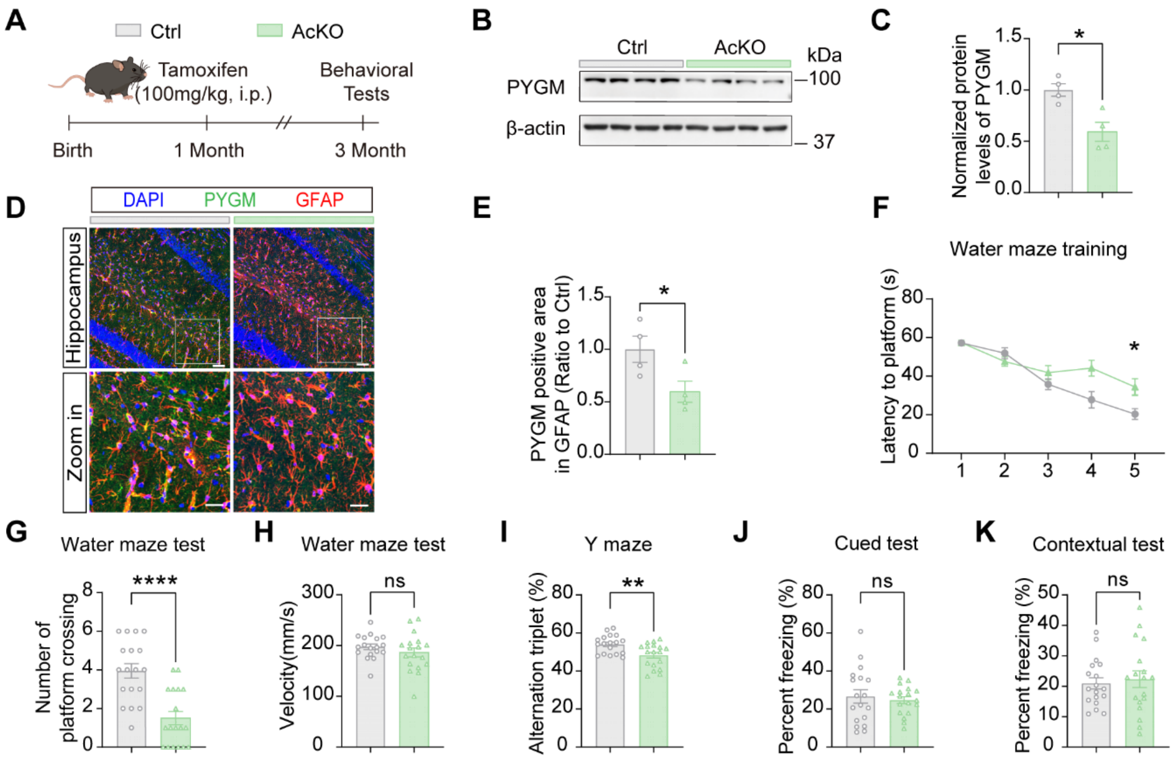

Figure S3. Astrocytic PYGM ablation impairs memory function in mice. (A) Experimental timeline for tamoxifen administration and behavioral testing in *Pygm*$^{flox/flox}$;*Aldh1L1*-cre^{ER} (AcKO) and *Pygm*$^{flox/flox}$ littermate control (Ctrl) male mice. (B, C) Immunoblot (B) and quantitative analysis (C) of PYGM protein levels in the hippocampus of AcKO and Ctrl mice. n = 4 per group. (D, E) Immunofluorescence staining of PYGM (green) and GFAP (red) (D) and their colocalization analysis (E) in brain sections from AcKO and Ctrl mice. The nuclei were counterstained with DAPI (blue). Scale bars: 100 μm; 50 μm for zoom-in images. n = 4 per group. (F-H) Mice were subjected to the Morris Water Maze test to compare the escape latency to reach the platform during a 5-day training phase (F), and the number of platform region crossings (G) and the average swimming speed (H) during the probe test. n = 18 mice per group. (I) Mice were compared

for their spontaneous alternations in the Y-maze test. n = 18 mice per group. (J, K) Mice were subjected to the fear conditioning test to compare the percentage of freezing time in the cued (J) and contextual (K) phases. n = 18 mice per group. Data are presented as mean ± SEM. *P* values were determined by two-tailed unpaired Student's *t* test in (C, E, and G-K), and two-way ANOVA followed by Dunn-Šídák post hoc analysis in (F). ns, not significant; *$P < 0.05$; **$P < 0.01$; ****$P < 0.0001$.

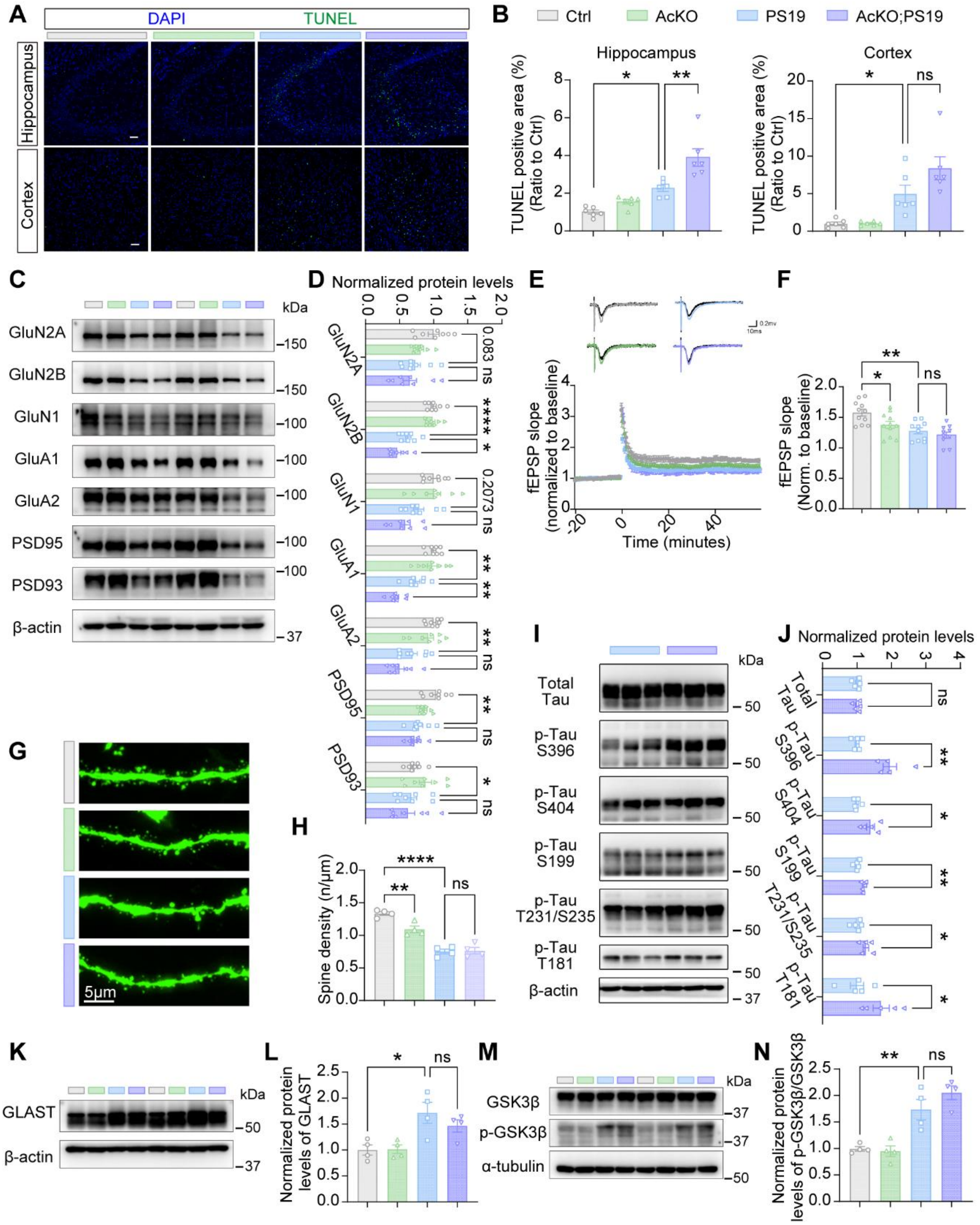

Figure S4. Astrocytic PYGM ablation impairs synaptic proteins and promotes tau pathologies. (A, B) TUNEL (green) and DAPI (blue) staining (A) and quantitative analysis of TUNEL staining (B) in brain sections from different mice**.** Scale bars: 100 µm. n = 6 per group. (C, D) Immunoblot (C) and quantitative analysis (D) of GluN2A, GluN2B, GluN1,

GluA1, GluA2, PSD95, and PSD93 protein levels in different mice. n = 6 per group. (E, F) Long-term potentiation (LTP) recordings in the hippocampal CA1 region (E) and quantitative analysis of the mean fEPSP slope during the final 10 minutes of recording (F) of different mice. n = 10-11 slices from 6 mice per group. (G, H) Representative confocal z-stack images of dendritic spines in the hippocampus of different mice (G) and quantitative analysis of dendritic spine density (H). Scale bar: 5 μm. n = 4 mice per group with 45-50 dendrites quantified in each group**.** (I, J) Immunoblot (I) and quantitative analysis (J) of total tau and phosphorylated tau at sites S396, S404, S199, T231/S235, and T181 in PS19 and AcKO;PS19 mice. n = 5 per group. (K, L) Immunoblot (K) and quantitative analysis (L) of GLAST protein levels in different mice. n = 4 per group. (M, N) Immunoblot (M) and quantitative analysis (N) of p-GSK3β and GSK3β protein levels in different mice. n = 4 per group. Data are presented as mean ± SEM. *P* values were determined by one-way ANOVA followed by Tukey's post hoc analysis in (B, D, F, H, L, and N), and two-tailed unpaired Student's *t* test in (J). ns, not significant; *$P < 0.05$; **$P < 0.01$; ****$P < 0.0001$.

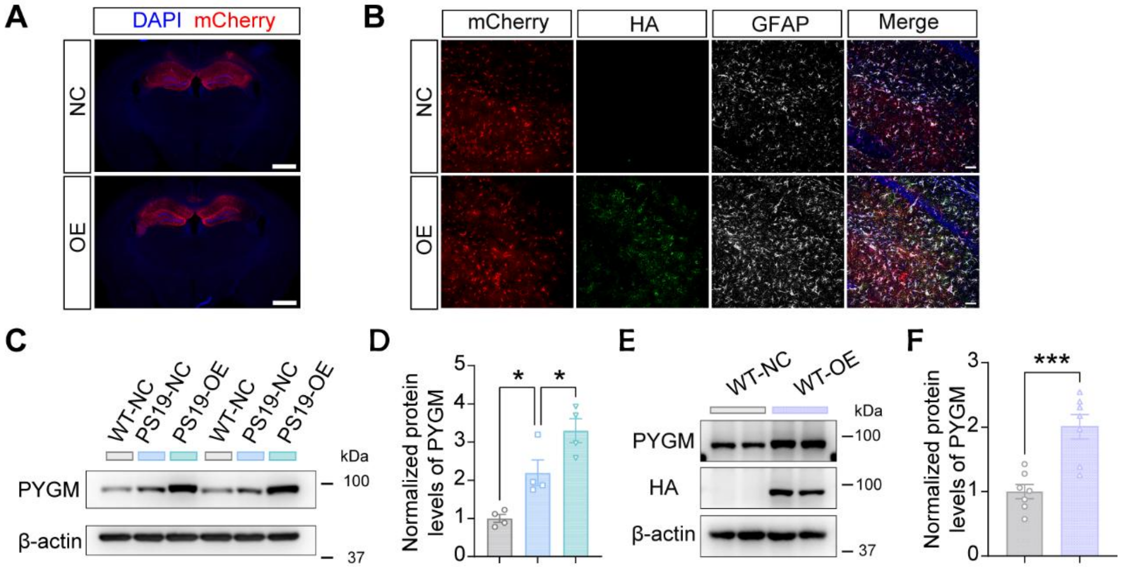

Figure S5. Validation of AAV-mediated PYGM overexpression in astrocytes. (A) Representative images of the hippocampal region from mice injected with AAV-*gfaABC1D-cherry* (NC, red) or AAV-*gfaABC1D-PYGM-HA-cherry* (OE, red). The nuclei were counterstained with DAPI (blue). Scale bars: 1000 μm. (B) Representative images showing co-immunofluorescence of mCherry (red), HA (green), and GFAP (white) in the hippocampus of mice injected with AAV-NC or AAV-OE. The nuclei were counterstained with DAPI (blue). Scale bars: 100 μm. (C, D) Immunoblot (C) and quantitative analysis (D) of PYGM protein levels in different mice. n = 4 per group. (E, F) Immunoblot (E) and quantitative analysis (F) of PYGM protein levels in PYGM-overexpressing WT mice. n = 7 per group. Data are presented as mean ± SEM. *P* values were determined by one-way ANOVA followed by Tukey's post hoc analysis in (D), and two-tailed unpaired Student's *t* test in (F). *$P < 0.05$; ***$P < 0.001$.

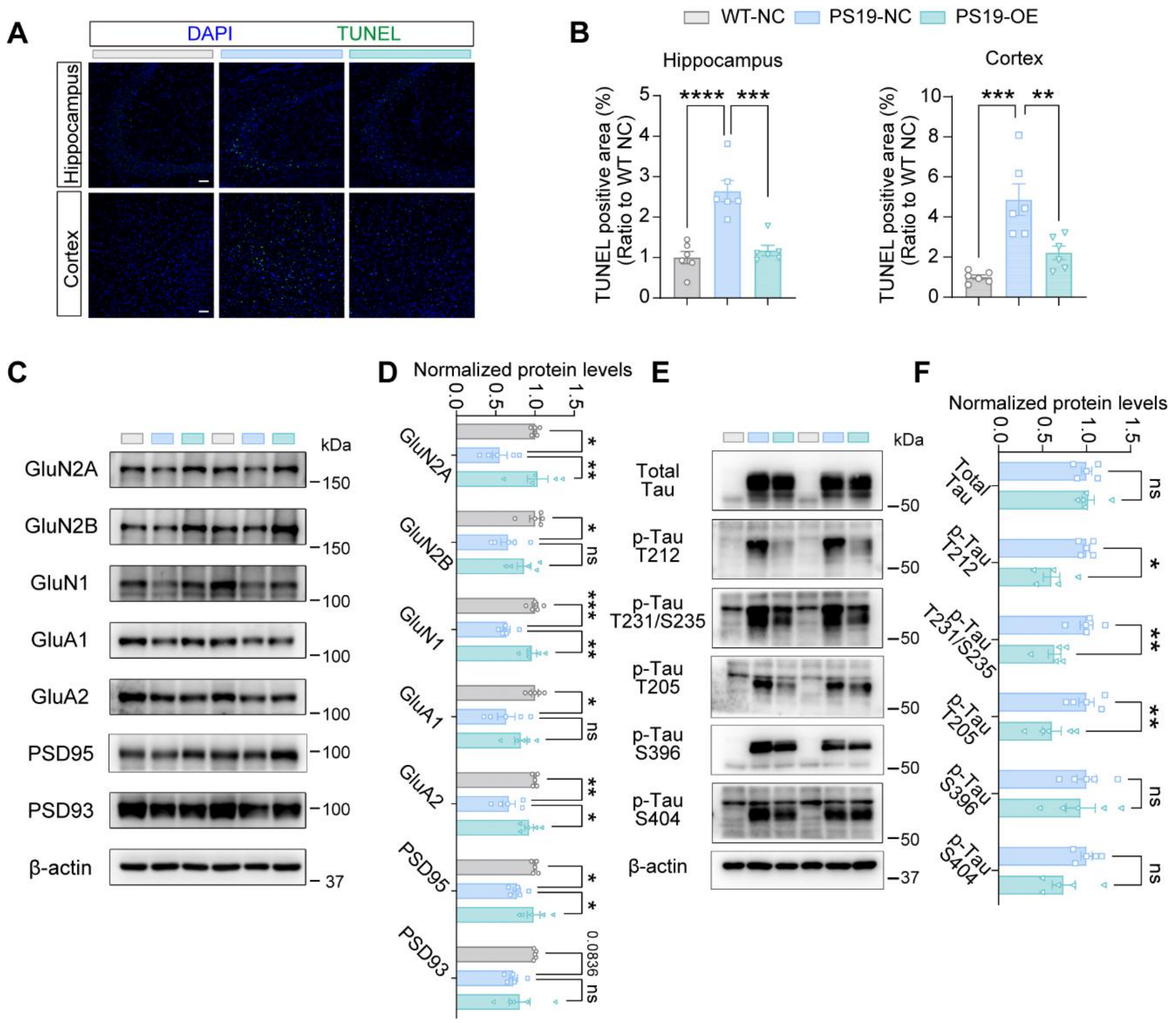

Figure S6. PYGM overexpression in astrocytes attenuates pathologies in PS19 mice. (A, B) TUNEL (green) and DAPI (blue) staining (A) and quantitative analysis of TUNEL staining (B) in brain sections from different mice**.** Scale bars: 100 μm. n = 6 per group. (C, D) Immunoblot (C) and quantitative analysis (D) of GluN2A, GluN2B, GluN1, GluA1, GluA2, PSD95, and PSD93 protein levels in different mice. n = 5 per group. (E, F) Immunoblot (E) and quantitative analysis (F) of total tau and phosphorylated tau at sites T212, T231/S235, T205, S396, and S404 in different mice. n = 5 per group. Data are presented as mean ± SEM. *P* values were determined by one-way ANOVA followed by Tukey's post hoc analysis in (B and D), and two-tailed unpaired Student's *t* test in (F). ns,

not significant; $*P < 0.05$; $**P < 0.01$; $***P < 0.001$; $****P < 0.0001$.

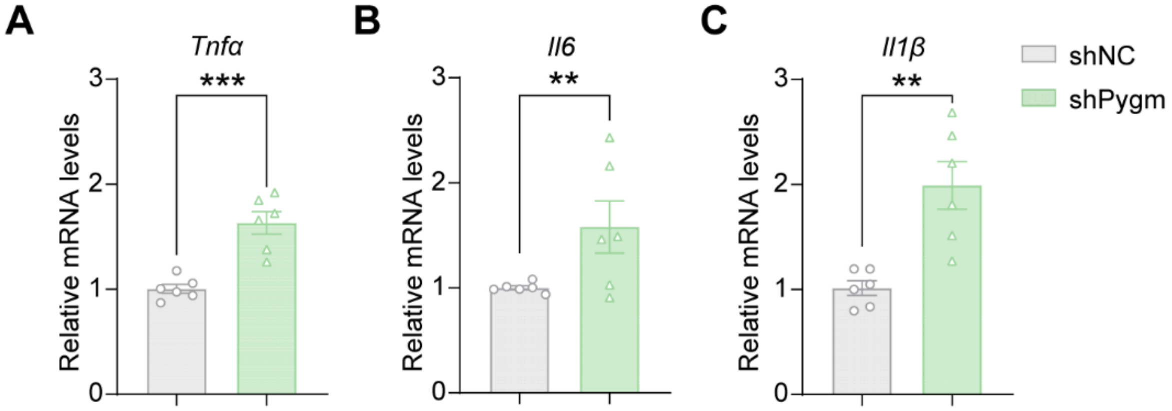

Figure S7. Astrocytic PYGM deficiency promotes the expression of pro-inflammatory factors. (A-C) Cultured primary astrocytes from WT mice were infected with lentiviruses expressing shRNA targeting *Pygm* (shPygm) or control shRNA (shNC). The mRNA levels of *Tnfα* (A), *Il6* (B), and *Il1β* (C) in shNC and shPygm astrocytes were analyzed by qRT-PCR for comparison. n = 6 experiments.

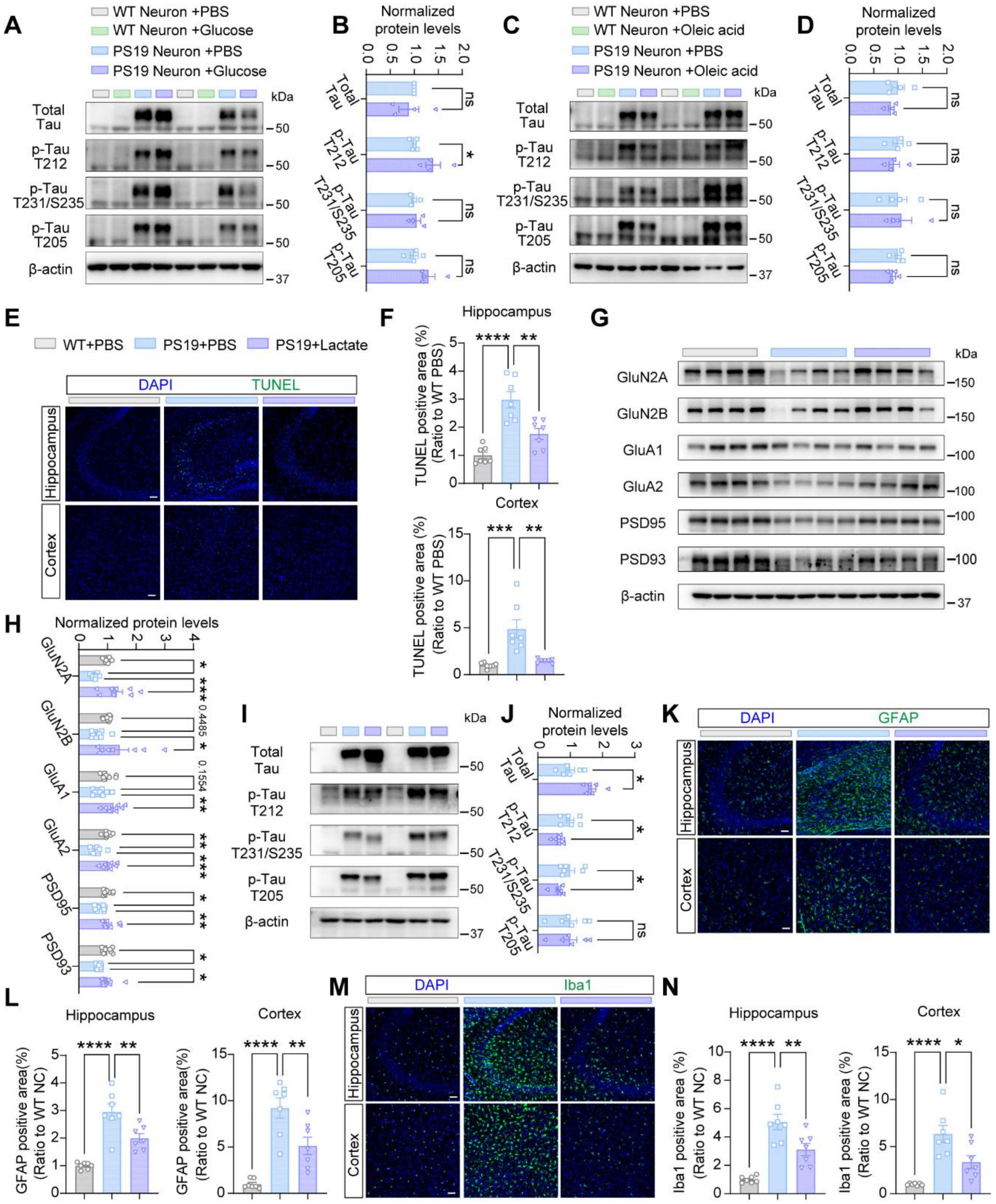

Figure S8. Lactate treatment attenuates pathologies in PS19 mice. (A, B) Immunoblot (A) and quantitative analysis (B) of total tau and phosphorylated tau at sites T212, T231/S235, and T205 in WT and PS19 neurons treated with PBS or D-glucose. n = 4 experiments. (C, D) Immunoblot (C) and quantitative analysis (D) of total tau and

phosphorylated tau at sites T212, T231/S235, and T205 in WT and PS19 neurons treated with PBS or Oleic acid. n = 4 experiments. (E, F) TUNEL (green) and DAPI (blue) staining (E) and quantitative analysis of TUNEL staining (F) in brain sections from different mice**.** Scale bars: 100 μm. n = 7 per group. (G, H) Immunoblot (G) and quantitative analysis (H) of GluN2A, GluN2B, GluA1, GluA2, PSD95, and PSD93 protein levels in different mice. n = 8 per group. (I, J) Immunoblot (I) and quantitative analysis (J) of total tau and phosphorylated tau at sites T212, T231/S235, and T205 in different mice. n = 6 per group. (K, L) Immunofluorescence staining of GFAP (green) (K) and quantitative analysis (L) in brain sections from different mice. The nuclei were counterstained with DAPI (blue). Scale bars: 100 μm. n = 7 per group. (M, N) Immunofluorescence staining of Iba1 (green) (M) and quantitative analysis (N) in brain sections from different mice. The nuclei were counterstained in DAPI (blue). Scale bars: 100 μm. n = 7 per group. Data are presented as the mean ± SEM. *P* values were determined by two-tailed unpaired Student's *t* test in (B, D, and J), and one-way ANOVA followed by Tukey's post hoc analysis in (F, H, L, and N). ns, not significant; *$P < 0.05$; **$P < 0.01$; ***$P < 0.001$; ****$P < 0.0001$.
